# Seasonality of confirmed malaria cases from 2008 to 2017 in Togo: a time series analysis by health district and target group

**DOI:** 10.1186/s12879-021-06893-z

**Published:** 2021-11-26

**Authors:** Anne Thomas, Tchaa A. Bakai, Tinah Atcha-Oubou, Tchassama Tchadjobo, Nadine Bossard, Muriel Rabilloud, Nicolas Voirin

**Affiliations:** 1 Université de Lyon, Lyon, France; 2 Université Lyon 1, Villeurbanne, France; 3 Service de Biostatistique et Bioinformatique, Pôle Santé Publique, Hospices Civils de Lyon, Lyon, France; 4 Équipe Biostatistique-Santé, Laboratoire de Biométrie et Biologie Évolutive, CNRS, UMR 5558, Villeurbanne, France; 5 Epidemiology and Modelling of Infectious Diseases (EPIMOD), Lent, France; 6 Programme National de Lutte contre le Paludisme (PNLP), Lomé, Togo

**Keywords:** Seasonality, Malaria, Seasonal-trend decomposition, National Malaria Control Programme, Togo

## Abstract

**Background:**

This study aimed to assess the seasonality of confirmed malaria cases in Togo and to provide new indicators of malaria seasonality to the National Malaria Control Programme (NMCP).

**Methods:**

Aggregated data of confirmed malaria cases were collected monthly from 2008 to 2017 by the Togo’s NMCP and stratified by health district and according to three target groups: children < 5 years old, children ≥ 5 years old and adults, and pregnant women. Time series analysis was carried out for each target group and health district. Seasonal decomposition was used to assess the seasonality of confirmed malaria cases. Maximum and minimum seasonal indices, their corresponding months, and the ratio of maximum/minimum seasonal indices reflecting the importance of malaria transmission, were provided by health district and target group.

**Results:**

From 2008 to 2017, 7,951,757 malaria cases were reported in Togo. Children < 5 years old, children ≥ 5 years old and adults, and pregnant women represented 37.1%, 57.7% and 5.2% of the confirmed malaria cases, respectively. The maximum seasonal indices were observed during or shortly after a rainy season and the minimum seasonal indices during the dry season between January and April in particular. In children < 5 years old, the ratio of maximum/minimum seasonal indices was higher in the north, suggesting a higher seasonal malaria transmission, than in the south of Togo. This is also observed in the other two groups but to a lesser extent.

**Conclusions:**

This study contributes to a better understanding of malaria seasonality in Togo. The indicators of malaria seasonality could allow for more accurate forecasting in malaria interventions and supply planning throughout the year.

**Supplementary Information:**

The online version contains supplementary material available at 10.1186/s12879-021-06893-z.

## Background

Malaria is an infectious disease caused by *Plasmodium* parasites that are transmitted to people through the bites of infected female *Anopheles* mosquitoes [1]. In 2019, the World Health Organization (WHO) estimated 215 million malaria cases and 348,000 malaria deaths for the African Region, which accounted for 94% of cases and deaths worldwide [2]. Of the five parasite species that cause malaria in humans, *Plasmodium falciparum* is the most prevalent in sub-Saharan region [1, 3]. In 2018, it was responsible for 99.7% of estimated malaria cases in the WHO African Region [4]. Children under 5 years old and pregnant women are at higher risk of adverse malaria outcomes such as severe malaria [5]. The most common manifestations of severe malaria in children are cerebral malaria or severe malarial anaemia [6]. Malaria deaths occurs mainly in African children under 5 years old. In 2019, 67% of total malaria deaths were estimated in children under 5 years old [2]. In pregnant women, *Plasmodium falciparum* infection increases the risk of maternal anaemia, miscarriage, stillbirth, foetal growth restriction, low birthweight, prematurity and neonatal mortality [7]. Despite the overall decline of malaria in Africa, malaria transmission is also characterised by spatial and temporal variations between and within countries [2, 3, 8].

The Global Technical Strategy for Malaria 2016–2030 report recommends that National Malaria Control Programmes (NMCP) analyse past malaria incidence data, risk factors related to the human host, parasites, vectors as well as the environment in order to inform national malaria control interventions [9]. Spatial and temporal analysis of malaria data can be a decision-making tool in order to better understand the heterogeneity of malaria transmission in a country, to guide malaria control programmes and to increase the efficiency of interventions [3, 10, 11]. Malaria transmission in West Africa, except for Algeria and Cabo Verde, is year-round with high seasonality in the Sahelian countries [2]. A conducive environment such as rainfall, high temperature and humidity is required for malaria transmission between the mosquito vector and its human host [12]. In the scientific literature, the seasonality of malaria is generally described by associating malaria data with climate data, instead of describing malaria seasonal patterns per se [13].

In Togo, the numbers of reported malaria cases and deaths were 2.4 million (99.0% of falciparum malaria) and 1275 in 2019, respectively [2]. For the last 15 years, large-scale interventions have been implemented by Togo’s NMCP to prevent malaria in target groups. For example, long-lasting insecticidal nets (LLINs) are distributed in routine medical visits among pregnant women and children under 1 year old, intermittent preventive treatment during pregnancy is provided, and a distribution of seasonal malaria chemoprevention (SMC) is implemented from July to September or October in children aged 3–59 months in the three northern regions of Togo (Savanes, Kara, Centrale). Malaria time series analysis is scarce in Togo. In 2012, Landoh et al*.* published a study on malaria morbidity and mortality from 2005 to 2010 in Est Mono district, Togo [14]. Most recently, Bakai et al*.* investigated the trend of reported malaria cases and deaths from 2008 to 2017 in the six health regions of Togo [15]. These two studies briefly addressed the seasonality of malaria but did not analyse it in-depth.

This study aimed to assess the seasonality of confirmed malaria cases from 2008 to 2017 by health district and target group in Togo, and to provide new indicators of malaria seasonality to the NMCP in order to avoid stock-outs of malaria commodities in health facilities during high malaria transmission.

## Methods

### Setting

**Fig. 1:**
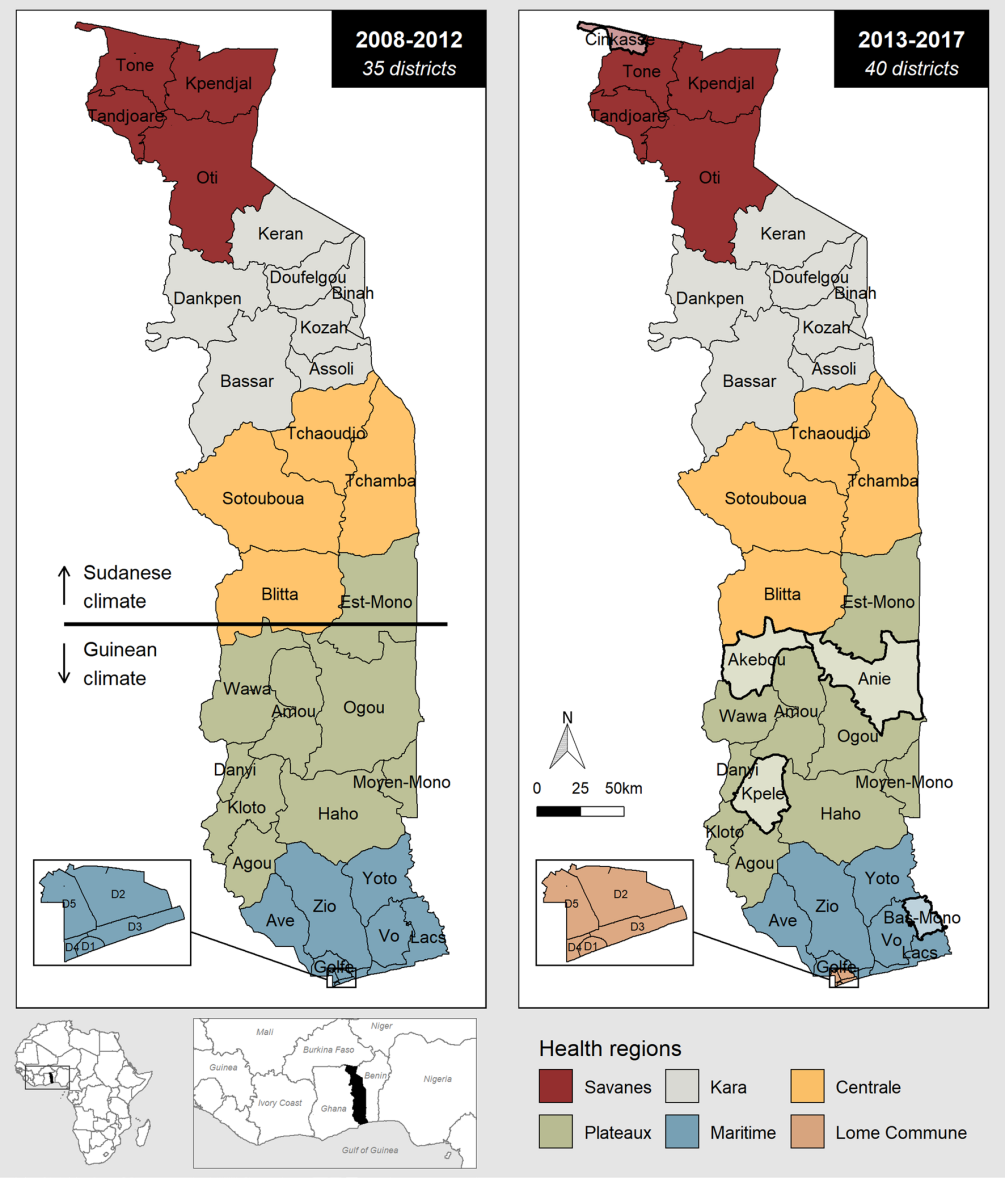
Health maps of Togo during the study period, 2008–2017

Togo is a West African country with a total area of 56,785 km^2^ [16] and 8.08 million inhabitants in 2019 [17]. The Togolese health system has a pyramidal structure with three levels: the Central Level which includes the NMCP, the Regional Level which includes health regions and the Peripheral Level which includes health districts and peripheral care units. From 2008 to 2012, Togo had five health regions (from north to south: Savanes, Kara, Centrale, Plateaux, Maritime) and 35 health districts. From 2013 to 2017, Togo had six health regions (creation of Lome-commune) and 40 health districts. Figure 1 shows the health maps of Togo during the study period. Togo has a tropical climate and is subdivided into two climate zones from the 8th parallel [18]. In the north, the climate is Sudanese type with a long rainy season from May to October. In the south, the climate is Guinean type, characterised by two rainy seasons from April to July and September–October.

### Data source and definition

Aggregated data of confirmed malaria cases collected routinely by the NMCP of Togo were analysed in this study. Data were collected monthly from January 2008 to December 2017 and stratified by health district and according to three target groups: (1) children < 5 years old, (2) children ≥ 5 years old and adults (without pregnant women), and (3) pregnant women. A detailed description of the Togolese health system and the routine collection of these data is presented elsewhere [15]. A confirmed malaria case is defined as a person with fever or history of fever (temperature ≥ 38.0 °C) over the past 2 days and who has a positive malaria diagnostic test by microscopy or rapid diagnostic test (RDT).

### Statistical analysis

Observed time series showed the presence of two artificial peaks in September 2012 and September 2016, due to an increase in the number of active screenings during one-off campaigns. An imputation by spline interpolation was performed to replace the values of these two artificial peaks (Additional file 1: Fig. S1). Time series analysis was carried out for the three target groups and the 35 health districts as defined during the period 2008–2012 (total of 105 time series). For each time series, the change of the number of confirmed malaria cases according to the month of the year during the period 2008–2017 was described using box plots.

A decomposition procedure based on locally-weighted regressions, known as STL decomposition [19], was used to decompose the time series into seasonal, trend-cycle and irregular components in order to assess the yearly seasonality of the number of confirmed malaria cases. The multiplicative model was used for all the time series. The mean absolute error (MAE) was in favour of a better fitting of the multiplicative model in comparison to the additive model for 86 of the 105 time series [20] (Additional file 1: Table S1). For the other 19 time series, the MAE difference between the two models was small. The multiplicative model is written as follows:

$${Y}_{t}={T}_{t}\times {S}_{t}\times {e}_{t}\Leftrightarrow {\mathrm{log}(Y}_{t})=\mathrm{log}({T}_{t})+\mathrm{log}({S}_{t})+\mathrm{log}({e}_{t})$$

${Y}_{t}$ is the number of confirmed malaria cases at time t; ${T}_{t}$, ${S}_{t}$ and ${e}_{t}$ are the trend-cycle, the seasonal and the irregular components at time t, respectively. This model assumes that the seasonal component is constant from year to year. The stationarity of residuals was checked using autocorrelograms and the Augmented Dickey–Fuller test. These two methods showed no deviation from the stationarity assumption of the model for each time series (Additional file 1: Fig. S2).

The seasonal component is composed of 12 values called seasonal indices, estimated by the exponential of $\mathrm{log}({S}_{t})$, that is ${S}_{t}$. Each value corresponds to a month of the year and is a multiplicative factor of the seasonally adjusted time series. Time series without seasonal variation have a seasonal index of 1. For each health district and target group, several indicators of malaria seasonality were extracted from the seasonal component: the maximum and minimum seasonal indices and their corresponding months, and the ratio of the maximum seasonal index to the minimum seasonal index. This ratio allows to quantify the amplitude of the variation in the number of confirmed malaria cases between the month with the minimum seasonal index and the month with the maximum seasonal index. It can be used as an indicator of the importance of malaria transmission.

A sensitivity analysis was undertaken to assess the effect of alternative imputation strategies on malaria seasonality indicators. The decomposition procedure was performed using the original time series (no imputation) and the time series after imputation of the values of the two artificial peaks by last observation carried forward.

All statistical analyses were performed using R software version 4.0.3 [21]. The decomposition procedure was carried out using the *stl* function of the *stats* package.

## Results

### Confirmed malaria cases

From 2008 to 2017, 7,951,757 malaria cases were reported in Togo (Table 1). Confirmed malaria cases increased from 291,362 cases in 2008 to 1,204,192 cases in 2017. Children < 5 years old, children ≥ 5 years old and adults, and pregnant women represented 37.1%, 57.7% and 5.2% of the confirmed malaria cases, respectively. The median number of confirmed malaria cases ranged from 12,480 in March to 36,832 in July in children < 5 years old, from 18,595 in March to 44,270 in November in children ≥ 5 years old and adults, and from 2112 in March to 4040 in August in pregnant women (Additional file 1: Fig. S3).

**Table 1:** Confirmed malaria cases in Togo from 2008 to 2017 by target group

Year	Children < 5 years old	Children ≥ 5 years old and adults	Pregnant women	Overall
2008	78,905	190,445	22,012	291,362
2009	126,783	241,065	25,058	392,906
2010	253,842	339,244	33,736	626,822
2011	217,376	285,424	30,401	533,201
2012	247,266	307,300	30,254	584,820
2013	376,230	495,782	43,105	915,117
2014	423,688	717,791	56,329	1,197,808
2015	425,733	626,681	54,003	1,106,417
2016	375,929	664,753	58,430	1,099,112
2017	420,967	723,114	60,111	1,204,192
Total	2,946,719	4,591,599	413,439	7,951,757

### Description of time series

**Fig. 2:**
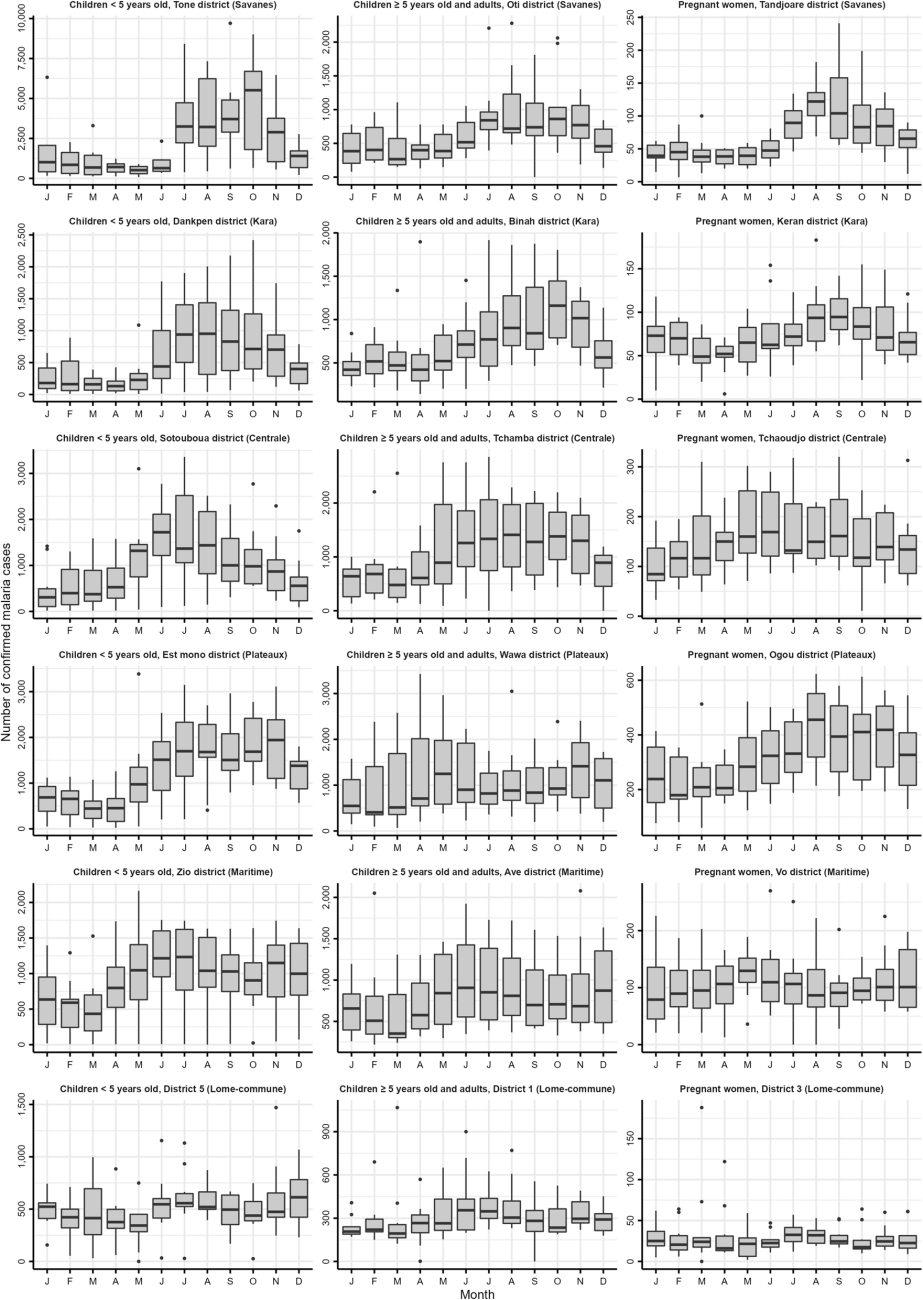
Change of the number of confirmed malaria cases according to the month of the year during the period 2008–2017 in Togo. The box plots represent the distribution of the number of cases for the decade studied, stratified by month. Example of 18 time series in the three target groups. Time series for children < 5 years old, for children ≥ 5 years old and adults, and for pregnant women are presented in the first, second and third columns, respectively. Time series have different Y-axis scales

All health districts had an annual seasonal component with the exception of certain districts such as those in the Lome-commune region (Fig. 2, Additional file 1: Fig. S4). The number of confirmed malaria cases was in median higher during or at the end of the rainy season (from May to October in the north and from April to July and September–October in the south). In the Tone, Oti and Tandjoare districts of the Savanes region presented in Fig. 2, the median numbers of confirmed malaria cases were higher in October in children < 5 years old (median: 5517; interquartile range (IQR): 1805–6696), in October in children ≥ 5 years old and adults (median: 862; IQR: 614–1033) and in August in pregnant women (median: 122; IQR: 101–136). Greater variability between years is often noted for the months with the highest median numbers of cases. For example, in children < 5 years old of the Est mono district, the IQR of confirmed malaria cases was 1104 to 2385 in November (month with a high median: 1943), while it was 229 to 607 in March (month with a low median: 444).

### Seasonal component of time series

**Fig. 3:**
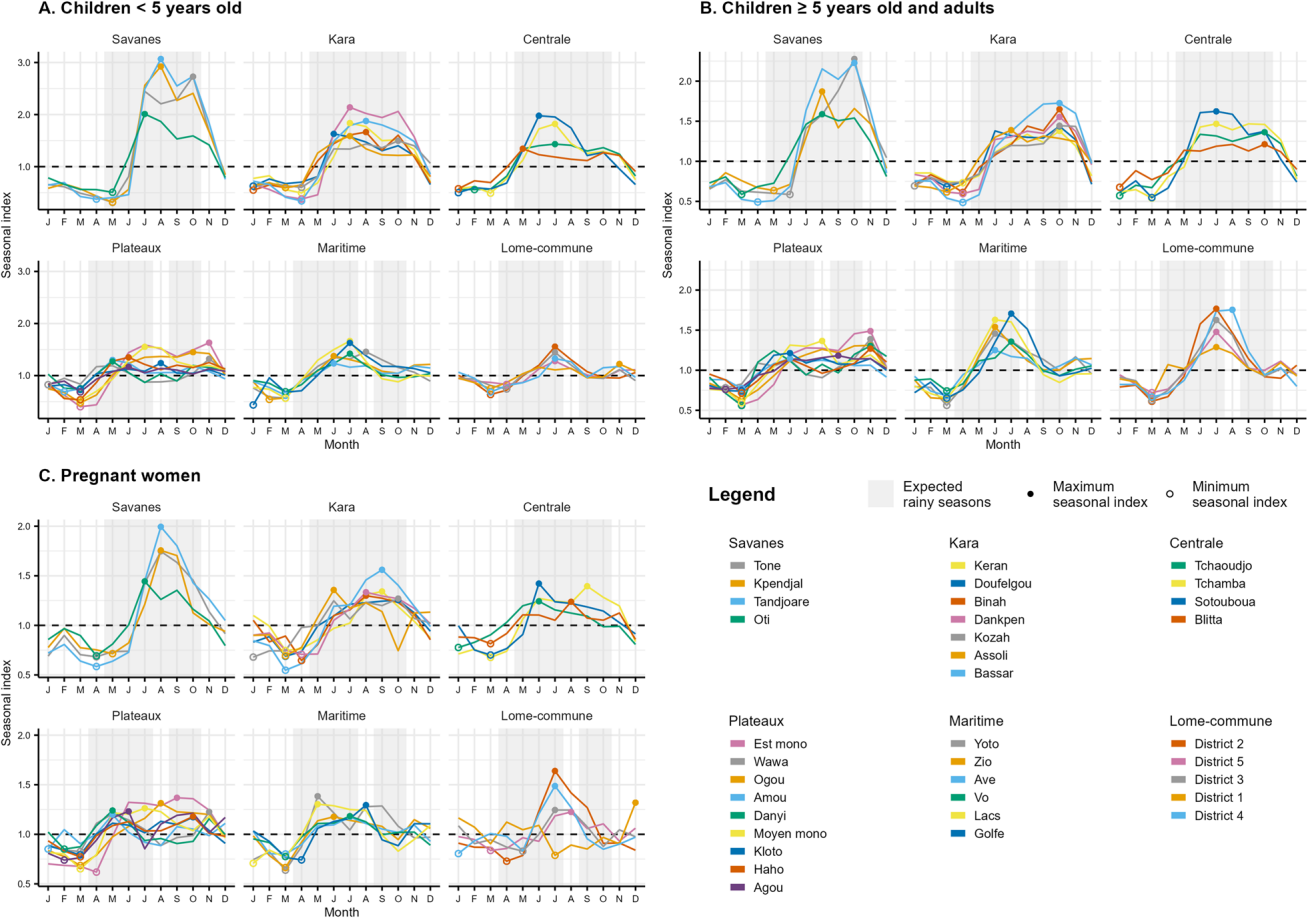
Seasonal component estimated by the decomposition procedure for each health district in children < 5 years old (**A**), in children ≥ 5 years old and adults (**B**), and in pregnant women (**C**). Filled dots indicate maximum seasonal indices and empty dots indicate minimum seasonal indices. Target groups have different Y-axis scales

The maximum seasonal indices were observed during or shortly after a rainy season and the minimum seasonal indices during the dry season between January and April in particular (Fig. 3, Table 2). In children < 5 years old, the maximum seasonal indices were observed between May and November, mainly in June, July and August (for 26 of the 35 health districts) and ranged from 1.17 to 3.07 in the Agou and Tandjoare districts, respectively. For the districts of the Savanes, Kara and Centrale regions, malaria transmission was highest in the middle of the single rainy season (July–August), while it was highest at the end of the first rainy season (June–July) in the southern regions. The minimum seasonal indices were observed between January and May, mainly in March and April (for 23 health districts) and ranged from 0.31 to 0.83 in the Tone district and the District 5, respectively. In children ≥ 5 years old and adults, the maximum seasonal indices were observed between June and November, mainly in October for the districts of the Savanes, Kara and Centrale regions, in November for the districts of the Plateaux region, and in June or July for the districts of the Maritime and Lome-commune regions. Thus, the highest malaria transmission was mainly at the end of rainy season (October for the northern regions and July for the southern regions). The maximum seasonal indices ranged from 1.15 to 2.28 in the Amou and Tone districts, respectively. In this population, the minimum seasonal indices were observed in March for 23 health districts and from January to June for the other districts. The minimum seasonal indices ranged from 0.49 to 0.78 in the Tandjoare and Wawa districts, respectively. In pregnant women, the maximum seasonal indices were observed between May and December and the minimum seasonal indices were observed between January and July, mainly in March and April (for 25 health districts). The influence of rainy season on the number of confirmed malaria cases among pregnant women was less evident than in other target groups. The maximum seasonal indices ranged from 1.17 to 1.99 in the Kloto and Tandjoare districts, respectively. The minimum seasonal indices ranged from 0.55 to 0.85 in the Bassar and Danyi districts, respectively. In children < 5 years old, the ratio of maximum/minimum seasonal indices was higher in the health districts of the northern regions than the southern regions. In the three northern regions, the number of confirmed malaria cases was multiplied by 2.31 and up to 9.12 between the month with the minimum seasonal index and the month with the maximum seasonal index. The amplitudes were lower in the three southern regions with a multiplicative factor between 1.54 and 4.07. This is also observed in the other two groups but to a lesser extent. For most of the health districts, this ratio was higher in children < 5 years old than in children ≥ 5 years old and adults or pregnant women.

**Table 2:** Indicators of malaria seasonality by health district and target group, extracted from the seasonal component estimated by the decomposition procedure

Health region	Health district	Latitude	Children < 5 years old					Children ≥ 5 years old and adults					Pregnant women				
			Month of max. SI	Month of min. SI	Max. SI	Min. SI	Ratio of max. SI/min. SI	Month of max. SI	Month of min. SI	Max. SI	Min. SI	Ratio of max. SI/min. SI	Month of max. SI	Month of min. SI	Max. SI	Min. SI	Ratio of max. SI/min. SI
Savanes	Tone	10.92	October	May	2.73	0.31	8.81	October	June	2.28	0.59	3.86	August	April	1.75	0.68	2.57
	Kpendjal	10.82	August	May	2.92	0.32	9.12	August	May	1.87	0.64	2.92	August	May	1.76	0.71	2.48
	Tandjoare	10.65	August	April	3.07	0.38	8.08	October	April	2.23	0.49	4.55	August	April	1.99	0.58	3.43
	Oti	10.33	July	May	2.01	0.51	3.94	August	March	1.59	0.59	2.69	July	April	1.44	0.69	2.09
Kara	Keran	10.04	July	April	1.84	0.50	3.68	October	April	1.38	0.73	1.89	September	March	1.34	0.75	1.79
	Doufelgou	9.81	June	January	1.63	0.63	2.59	October	March	1.44	0.68	2.12	October	March	1.26	0.69	1.83
	Binah	9.77	August	January	1.66	0.55	3.02	October	April	1.65	0.61	2.70	August	April	1.30	0.65	2.00
	Dankpen	9.70	July	April	2.14	0.38	5.63	October	April	1.55	0.60	2.58	August	April	1.33	0.71	1.87
	Kozah	9.55	October	April	1.50	0.60	2.50	October	January	1.44	0.69	2.09	October	January	1.27	0.68	1.87
	Assoli	9.34	July	March	1.59	0.60	2.65	July	March	1.39	0.62	2.24	June	March	1.36	0.69	1.97
	Bassar	9.27	August	April	1.88	0.34	5.53	October	April	1.73	0.49	3.53	September	March	1.56	0.55	2.84
Centrale	Tchaoudjo	9.00	July	February	1.43	0.56	2.55	October	January	1.36	0.57	2.39	June	January	1.24	0.78	1.59
	Tchamba	8.75	July	March	1.82	0.49	3.71	July	March	1.47	0.54	2.72	September	March	1.39	0.68	2.04
	Sotouboua	8.66	June	January	1.98	0.50	3.96	July	March	1.62	0.55	2.95	June	March	1.42	0.70	2.03
	Blitta	8.16	May	January	1.34	0.58	2.31	October	January	1.21	0.68	1.78	August	March	1.24	0.82	1.51
Plateaux	Est mono	8.12	November	March	1.63	0.40	4.07	November	March	1.49	0.57	2.61	September	April	1.37	0.62	2.21
	Wawa	7.67	November	January	1.32	0.82	1.61	November	February	1.39	0.78	1.78	November	March	1.23	0.83	1.48
	Ogou	7.57	October	March	1.45	0.48	3.02	November	March	1.31	0.63	2.08	August	March	1.31	0.69	1.90
	Amou	7.55	May	February	1.30	0.69	1.88	June	March	1.15	0.77	1.49	May	January	1.20	0.85	1.41
	Danyi	7.25	May	March	1.27	0.75	1.69	November	March	1.30	0.56	2.32	May	February	1.24	0.85	1.46
	Moyen mono	7.18	July	March	1.55	0.52	2.98	August	March	1.37	0.59	2.32	July	March	1.26	0.65	1.94
	Kloto	7.05	August	March	1.24	0.73	1.70	June	March	1.21	0.74	1.64	October	March	1.17	0.79	1.48
	Haho	7.02	June	March	1.35	0.54	2.50	November	March	1.27	0.72	1.76	October	March	1.18	0.77	1.53
	Agou	6.76	June	March	1.17	0.69	1.70	September	February	1.18	0.76	1.55	June	February	1.23	0.74	1.66
Maritime	Yoto	6.68	August	March	1.46	0.57	2.56	June	March	1.46	0.56	2.61	May	March	1.38	0.64	2.16
	Zio	6.54	June	February	1.38	0.54	2.56	June	March	1.54	0.64	2.41	June	March	1.18	0.66	1.79
	Ave	6.46	June	March	1.23	0.62	1.98	June	March	1.25	0.68	1.84	July	March	1.18	0.80	1.47
	Vo	6.38	July	March	1.42	0.69	2.06	July	March	1.36	0.74	1.84	July	March	1.18	0.78	1.51
	Lacs	6.38	July	March	1.66	0.56	2.96	June	March	1.63	0.65	2.51	May	January	1.30	0.71	1.83
	Golfe	6.22	July	January	1.63	0.44	3.70	July	March	1.71	0.65	2.63	August	April	1.29	0.74	1.74
Lome-commune	District 2	6.18	July	March	1.56	0.64	2.44	July	March	1.77	0.61	2.90	July	April	1.64	0.73	2.25
	District 5	6.17	July	April	1.28	0.83	1.54	July	March	1.48	0.72	2.06	August	March	1.22	0.84	1.45
	District 3	6.15	July	April	1.45	0.74	1.96	July	March	1.63	0.62	2.63	July	May	1.24	0.83	1.49
	District 1	6.13	November	March	1.22	0.75	1.63	July	March	1.29	0.66	1.95	December	July	1.32	0.79	1.67
	District 4	6.13	July	March	1.33	0.68	1.96	August	March	1.75	0.67	2.61	July	January	1.49	0.81	1.84

The maximum and minimum seasonal indices and their corresponding months, and the ratio of the maximum seasonal index to the minimum seasonal index are presented. Health districts were classified according to their latitude

max. SI, maximum seasonal index; min. SI, minimum seasonal index

### Trend considerations

**Fig. 4:**
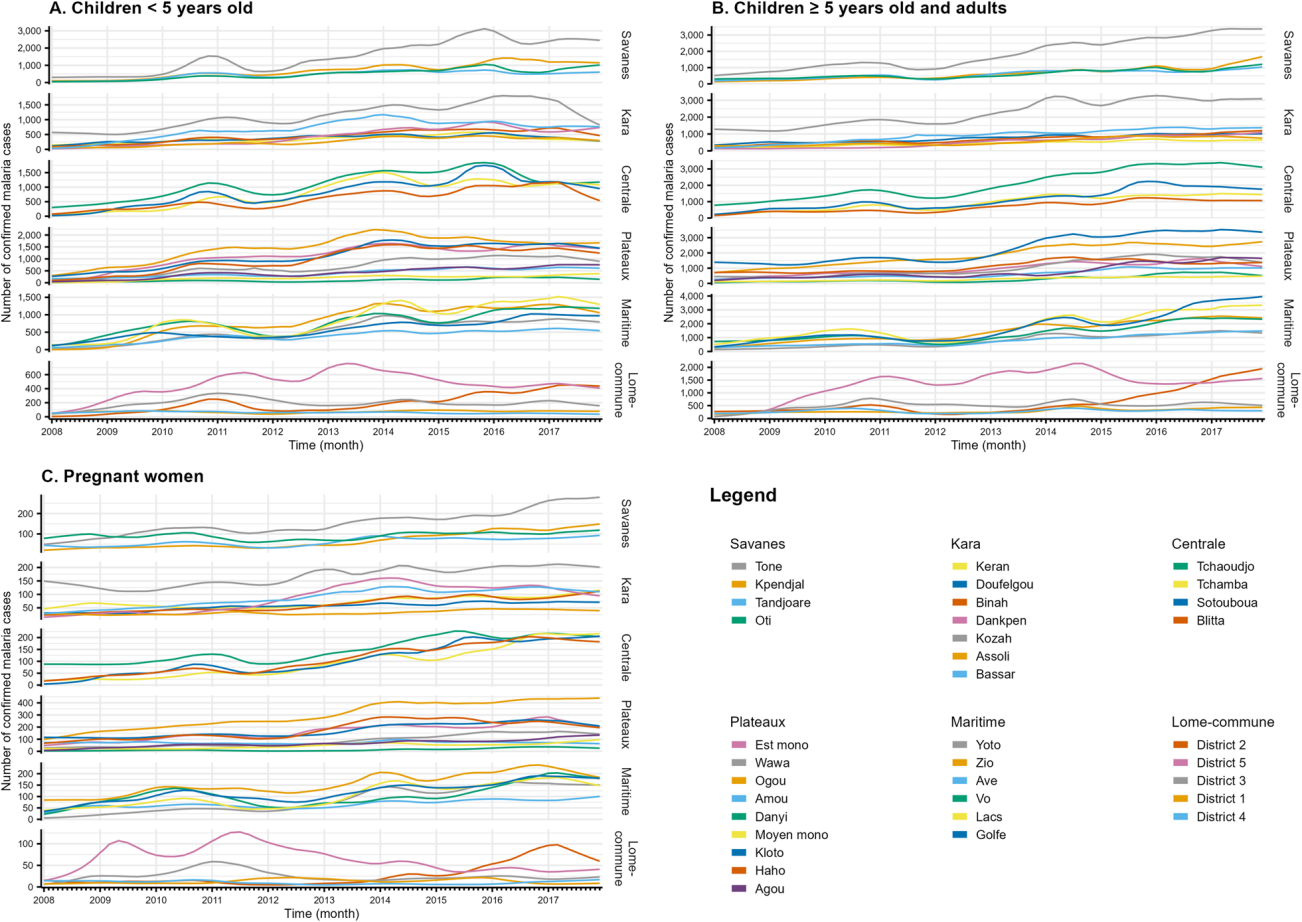
Trend-cycle component estimated by the decomposition procedure for each health district in children < 5 years old (**A**), in children ≥ 5 years old and adults (**B**), and in pregnant women (**C**). Health regions and target groups have different Y-axis scales

A trend of increasing confirmed malaria cases over time was observed in most health districts (Fig. 4). In the children < 5 years old, the number of confirmed malaria cases provided by the trend-cycle component ranged from 2 to 571 in January 2008 and from 37 to 2448 in December 2017 according to the district. In children ≥ 5 years old and adults, the number of confirmed malaria cases ranged from 54 to 1389 in January 2008 and from 298 to 3961 in December 2017. In pregnant women, the number of confirmed malaria cases ranged from 3 to 149 in January 2008 and from 8 to 439 in December 2017 according to the district.

### Sensitivity analysis

The sensitivity analysis did not show major differences in comparison to the results of the main analysis (Additional file 1: Fig. S5). However, the months of the maximum or minimum values of the estimated seasonality parameters were different for some time series, with a difference of only 1 month before or after.

## Discussion

To our knowledge, this analysis investigated for the first time the seasonality of confirmed malaria cases from 2008 to 2017 in Togo, by health district and target group. This study contributes to a better understanding of malaria epidemiology in Togo based on data from the NMCP.

As expected, the number of confirmed malaria cases increased during or shortly after a rainy season. Malaria is a vector-borne disease and *Anopheles* mosquitoes are sensitive to weather conditions. Rainfall is one of the known determinants of the malaria seasonality in the scientific literature, as well as high temperature [13]. In tropical and holoendemic area in Ghana, Krefis et al*.* found a strong temporal association between rainfall and malaria incidence in 1993 children < 15 years old and encouraged the development of early warning systems using high-resolution precipitation data to forecast malaria incidence in highly endemic area [22]. Seasonal variations of malaria were shown throughout the whole country in Togo, even if they were less evident in some districts of Lome-commune region. Malaria transmission is more intense in the northern health districts than in the southern health districts, especially in children < 5 years old. A study conducted by Diouf et al*.* used the Liverpool Malaria Model to explore seasonal and long-term trends with simulated malaria data and used observed malaria cases provided by the NMCP of Senegal for model validation [23]. They concluded that malaria transmission in West Africa occurs toward the end of the rainy season, following heavy and frequent monsoon rains. Malaria transmission also seems to follow the latitudinal rainfall gradient in West Africa, as shown in the present study. Although a link between rainy seasons and malaria seasonality seems to be emerging, other factors may influence malaria transmission such as temperature, topography of health districts, malaria control activities. For example, monthly rainfall amounts or the distribution of SMC in children aged 3–59 months from July for 3–4 months in the three northern regions of Togo (since 2013 in the Savanes region and since 2016 in the Kara and Centrale regions) could help in the interpretation of malaria seasonality in Togo. Further analysis of the factors associated with malaria seasonality in Togo is needed and will be the subject of a future study.

Furthermore, this analysis reported an increase in confirmed malaria cases over time in most health districts and target groups. This result is consistent with two studies carried out in Togo, probably due to better registration of malaria cases and the deployment of community health workers (CHWs) at national level along time through increased international financial and technical resources [14, 15]. CHWs were trained to use RDTs to diagnose malaria in the communities and to treat confirmed malaria cases. Widespread deployment of CHWs led to better diagnosis of malaria cases and therefore an increase in registered cases [15]. Another time series analysis conducted in the Democratic Republic of Congo from 2005 and 2014 showed an increase in confirmed malaria cases associated with the introduction of RDTs [24]. In a spatiotemporal analysis of malaria in the capital of Burkina Faso, malaria incidence also increased over the 2011–2015 study period [25].

These findings have important implications for the NMCP of Togo. Firstly, the programme does not have tools that take into account malaria seasonality in stock management so far. The quantification of malaria commodities, such as artemisinin-based combination therapies (ACTs), artesunate injectable, sulfadoxine–pyrimethamine/amodiaquine, RDTs and LLINs, is carried out at the national level and they are stocked at the Procurement Centre of Essential and Generic Drugs (CAMEG) and at regional supply pharmacies. Each quarter, health districts are supplied according to their needs, based on malaria cases from the previous quarter. However, it is possible to request more malaria commodities (antimalarial treatments, RDTs) when the rainy season arrives. Health districts always have a 3-month safety stock, which is rebuilt with the next order. These new indicators of malaria seasonality by health district and target group could allow for more accurate forecasting in malaria interventions and supply planning by Togo’s NMCP throughout the year and ensure the availability of malaria drugs and diagnostic tests to beneficiaries in each health facility. The ratio of maximum/minimum seasonal indices, interpreted as a multiplicative factor of the number of confirmed malaria cases between the month with the minimum seasonal index and the month with the maximum seasonal index, may be a useful indicator to describe the intensity of malaria transmission and enable a better quantification of the supply of malaria commodities in order to avoid stocks-out of these commodities during periods of high malaria transmission. Secondly, this analysis can help the NCMP to guide its malaria control interventions in specific areas or target groups, and to reduce the intensity of malaria transmission during the rainy season. For example, in health districts of high and seasonal malaria transmission, long-acting ACTs could be recommended to limit the resurgence of malaria cases as mentioned by Cairns et al. [26]. Moreover, the distribution of SMC in children aged 3–59 months could be extended to certain districts of the Plateaux region that meet the criteria defined by the WHO for the implementation of this malaria control activity [27]. Confirmed malaria cases in the Est mono and Ogou districts occurred mainly during the rainy season (Additional file 1: Fig. S4) and their ratio of maximum/minimum seasonal indices were among the highest in the Plateaux region with values of 4.07 and 3.02, respectively, reflecting a high seasonal malaria transmission (Table 2). Further analysis is probably needed to discuss the eligibility of these districts for SMC deployment.

This analysis has some limitations. On the one hand, the stratified analysis by health district did not take into account the new delimitation of large health districts since 2013 in order to estimate a 10-year average seasonality. These territories that have been split into two health areas (Fig. 1) have common malaria seasonality indicators, which may pose a challenge for a precise programmatic approach. On the other hand, data were collected and aggregated by target group, and data on age were not provided. Although the “Children ≥ 5 years old and adults” group is very heterogenous, it was not possible to split it into several subgroups. Children, in whom immunity to malaria is being acquired through repeated exposure to parasites [28], and adults are not at the same risk of malaria. The decline in the level of malaria transmission in sub-Saharan Africa [29] is leading to a later acquisition of immunity to malaria than previously, and uncomplicated and severe malaria has become more prevalent in school-age children [30]. In this population, malaria can impair haemoglobin concentration, school performance, and cognitive functions caused by either cerebral malaria or by repeated episodes of uncomplicated malaria [30]. School-age children and teenagers are described as emerging populations at risk by Nkumama et al*.* who recommended to extend control malaria strategies focused on children under 5 years old to older age groups [3]. Two recent meta-analyses published in 2020 showed that preventive treatment of malaria among African school-age children was associated with reductions of *Plasmodium falciparum* prevalence, anaemia and subsequent clinical malaria across transmission settings [31]. The burden of malaria in school-age children is unknown in Togo, due to the subdivision of the population into three target groups. A better understanding of malaria prevalence in school-age children is needed to integrate this population into malaria control strategies.

The use of routine health information system (RHIS) data needs to be encouraged in a context of limited resources. In many low- and middle-income countries, as in Togo, the implementation of a web-based system called the district health information system 2 (DHIS-2) is a great opportunity to conduct scientific research, but RHIS data are still underused [32]. Nevertheless, progress has been made in the use of these data in the field of malaria using advanced statistical methods [32, 33].

## Conclusions

This first study on malaria seasonality in Togo provides several epidemiological insights. The number of confirmed malaria cases increased during or shortly after a rainy season. A higher seasonality was shown in the northern health districts than in the southern health district, especially in children < 5 years old. In addition, this study provides indicators of seasonality that can be useful to the NMCP of Togo in order to target interventions and avoid stocks-outs of malaria commodities during periods of high transmission. These first findings call for more in-depth knowledge of the seasonality of malaria, particularly its link with climatic data.

## Supplementary Information

**Additional file 1: Table S1.** Mean absolute error (MAE) of additive and multiplicative time series decomposition models by health district and target group. **Figure S1.** Description of confirmed malaria cases for each health district in children < 5 years old (A), in children ≥ 5 years old and adults (B), and in pregnant women (C) before and after imputation by spline interpolation, from 2008 to 2017 in Togo. Health regions and target groups have different Y-axis scales. **Figure S2.** Autocorrelograms of residuals estimated by the decomposition procedure for each health district in children < 5 years old (A), in children ≥ 5 years old and adults (B), and in pregnant women (C). Abbreviation: ACF, autocorrelation function. **Figure S3.** Change of the number of confirmed malaria cases according to the month of the year during the period 2008–2017 in Togo. The box plots represent the distribution of the number of cases for the decade studied, stratified by month. Data are grouped by target group. Time series have different Y-axis scales. **Figure S4.** Change of the number of confirmed malaria cases according to the month of the year during the period 2008-2017 in Togo. The box plots represent the distribution of the number of cases for the decade studied, stratified by month. Each health district in children < 5 years old (A), in children ≥ 5 years old and adults (B), and in pregnant women (C) are presented. Time series have different Y-axis scales. **Figure S5.** Results of different imputation strategies of the values of the two artificial peaks on seasonal component estimated by the decomposition procedure. Each health district in children < 5 years old (A), in children ≥ 5 years old and adults (B), and in pregnant women (C) are presented. Imputation by spline interpolation (main analysis), imputation by last observation carried forward, and no imputation (original data) corresponds to the grey, orange and red curves, respectively. Filled dots indicate maximum seasonal indices and empty dots indicate minimum seasonal indices. Time series have different Y-axis scales.

## Data Availability

The data analysed here are the property of the Togolese Ministry of Health and cannot be made available by the authors. To access the data, interested parties can get information from the Ministry of Health of Togo.

## Abbreviations

ACT: Artemisinin-based combination therapy
CAMEG: *Centrale d’achat des médicaments essentiels et génériques*
CHW: Community health worker
DHIS-2: District health information system 2
IQR: Interquartile range
LLIN: Long-lasting insecticidal net
MAE: Mean absolute error
NMCP: National malaria control programme
RDT: Rapid diagnostic test
RHIS: Routine health information system
SMC: Seasonal malaria chemoprevention
STL: Seasonal-trend decomposition procedure based on loess
WHO: World Health Organization
