# Supplementary material for: Seasonality of confirmed malaria cases from 2008 to 2017 in Togo: a time series analysis by health district and target group
Source: BMC Infect Dis. 2021 Nov 26;21:1189. doi: 10.1186/s12879-021-06893-z (PMC8620157; doi:10.1186/s12879-021-06893-z)
Supplement: Supplementary file 1 — Additional file 1: Table S1. Mean absolute error (MAE) of additive and multiplicative time series decomposition models by health district and target group. Figure S1. Description of confirmed malaria cases for each health district in children < 5 years old (A), in children ≥ 5 years old and adults (B), and in pregnant women (C) before and after imputation by spline interpolation, from 2008 to 2017 in Togo. Health regions and target groups have different Y-axis scales. Figure S2. Autocorrelograms of residuals estimated by the decomposition procedure for each health district in children < 5 years old (A), in children ≥ 5 years old and adults (B), and in pregnant women (C). Abbreviation: ACF, autocorrelation function. Figure S3. Change of the number of confirmed malaria cases according to the month of the year during the period 2008–2017 in Togo. The box plots represent the distribution of the number of cases for the decade studied, stratified by month. Data are grouped by target group. Time series have different Y-axis scales. Figure S4. Change of the number of confirmed malaria cases according to the month of the year during the period 2008-2017 in Togo. The box plots represent the distribution of the number of cases for the decade studied, stratified by month. Each health district in children < 5 years old (A), in children ≥ 5 years old and adults (B), and in pregnant women (C) are presented. Time series have different Y-axis scales. Figure S5. Results of different imputation strategies of the values of the two artificial peaks on seasonal component estimated by the decomposition procedure. Each health district in children < 5 years old (A), in children ≥ 5 years old and adults (B), and in pregnant women (C) are presented. Imputation by spline interpolation (main analysis), imputation by last observation carried forward, and no imputation (original data) corresponds to the grey, orange and red curves, respectively. Filled dots indicate maximum seasonal indices and e [file 12879_2021_6893_MOESM1_ESM.docx]

**Table S1: Mean absolute error (MAE) of additive and multiplicative time series decomposition models by health district and target group.**

|  |  | **Children < 5 years old** | | |  | **Children ≥ 5 years old and adults** | | |  | **Pregnant women** | | |
| --- | --- | --- | --- | --- | --- | --- | --- | --- | --- | --- | --- | --- |
| **Health region** | **Health district** | **Additive model** | **Multiplicative model** | **Best model** |  | **Additive model** | **Multiplicative model** | **Best model** |  | **Additive model** | **Multiplicative model** | **Best model** |
| Savanes | Tone | 874.3 | 601.3 | Multiplicative |  | 608.3 | 423.9 | Multiplicative |  | 45.9 | 37.0 | Multiplicative |
|  | Kpendjal | 326.1 | 304.6 | Multiplicative |  | 222.1 | 201.3 | Multiplicative |  | 16.3 | 15.3 | Multiplicative |
|  | Tandjoare | 223.0 | 163.2 | Multiplicative |  | 233.7 | 178.9 | Multiplicative |  | 14.1 | 12.5 | Multiplicative |
|  | Oti | 201.2 | 159.3 | Multiplicative |  | 164.2 | 148.5 | Multiplicative |  | 18.5 | 18.7 | Additive |
| Kara | Keran | 96.7 | 89.2 | Multiplicative |  | 127.4 | 109.8 | Multiplicative |  | 13.7 | 13.2 | Multiplicative |
|  | Doufelgou | 94.0 | 86.7 | Multiplicative |  | 153.6 | 139.5 | Multiplicative |  | 12.2 | 12.3 | Additive |
|  | Binah | 119.3 | 100.3 | Multiplicative |  | 157.9 | 138.2 | Multiplicative |  | 13.9 | 12.7 | Multiplicative |
|  | Dankpen | 205.1 | 128.2 | Multiplicative |  | 176.1 | 135.0 | Multiplicative |  | 18.1 | 16.5 | Multiplicative |
|  | Kozah | 277.4 | 276.2 | Multiplicative |  | 446.9 | 424.8 | Multiplicative |  | 30.0 | 30.4 | Additive |
|  | Assoli | 79.7 | 66.8 | Multiplicative |  | 128.3 | 118.7 | Multiplicative |  | 8.6 | 8.8 | Additive |
|  | Bassar | 195.5 | 174.8 | Multiplicative |  | 272.1 | 239.1 | Multiplicative |  | 17.8 | 17.2 | Multiplicative |
| Centrale | Tchaoudjo | 233.3 | 207.2 | Multiplicative |  | 374.3 | 327.7 | Multiplicative |  | 24.2 | 23.9 | Multiplicative |
|  | Tchamba | 260.0 | 194.8 | Multiplicative |  | 255.7 | 213.3 | Multiplicative |  | 19.2 | 15.4 | Multiplicative |
|  | Sotouboua | 286.8 | 273.3 | Multiplicative |  | 284.6 | 296.4 | Additive |  | 30.0 | 31.1 | Additive |
|  | Blitta | 154.7 | 140.4 | Multiplicative |  | 137.1 | 122.1 | Multiplicative |  | 22.4 | 20.6 | Multiplicative |
| Plateaux | Est mono | 288.9 | 251.3 | Multiplicative |  | 196.4 | 179.4 | Multiplicative |  | 30.2 | 31.2 | Additive |
|  | Wawa | 132.9 | 129.6 | Multiplicative |  | 236.3 | 238.0 | Additive |  | 14.5 | 14.9 | Additive |
|  | Ogou | 252.8 | 205.6 | Multiplicative |  | 323.3 | 280.0 | Multiplicative |  | 35.7 | 34.0 | Multiplicative |
|  | Amou | 75.0 | 69.9 | Multiplicative |  | 159.7 | 154.2 | Multiplicative |  | 14.3 | 14.3 | Additive |
|  | Danyi | 40.0 | 38.1 | Multiplicative |  | 106.1 | 105.5 | Multiplicative |  | 4.3 | 4.0 | Multiplicative |
|  | Moyen mono | 41.8 | 42.8 | Additive |  | 53.4 | 50.3 | Multiplicative |  | 8.2 | 7.8 | Multiplicative |
|  | Kloto | 151.0 | 142.2 | Multiplicative |  | 349.9 | 318.9 | Multiplicative |  | 20.8 | 20.6 | Multiplicative |
|  | Haho | 192.3 | 172.0 | Multiplicative |  | 201.2 | 194.3 | Multiplicative |  | 28.7 | 28.2 | Multiplicative |
|  | Agou | 82.5 | 80.2 | Multiplicative |  | 212.4 | 215.7 | Additive |  | 13.5 | 14.1 | Additive |
| Maritime | Yoto | 127.3 | 115.0 | Multiplicative |  | 169.1 | 154.3 | Multiplicative |  | 15.7 | 13.7 | Multiplicative |
|  | Zio | 147.3 | 126.6 | Multiplicative |  | 278.4 | 240.9 | Multiplicative |  | 26.8 | 26.9 | Additive |
|  | Ave | 67.8 | 62.3 | Multiplicative |  | 134.2 | 116.4 | Multiplicative |  | 9.2 | 9.1 | Multiplicative |
|  | Vo | 168.6 | 163.0 | Multiplicative |  | 288.3 | 271.3 | Multiplicative |  | 21.7 | 21.7 | Additive |
|  | Lacs | 202.5 | 187.2 | Multiplicative |  | 463.5 | 394.0 | Multiplicative |  | 19.4 | 18.1 | Multiplicative |
|  | Golfe | 128.7 | 118.8 | Multiplicative |  | 422.1 | 339.3 | Multiplicative |  | 27.7 | 27.7 | Additive |
| Lome-commune | District 2 | 75.4 | 70.7 | Multiplicative |  | 251.8 | 206.6 | Multiplicative |  | 15.8 | 13.8 | Multiplicative |
|  | District 5 | 115.7 | 114.5 | Multiplicative |  | 273.6 | 264.8 | Multiplicative |  | 28.2 | 24.9 | Multiplicative |
|  | District 3 | 51.8 | 51.3 | Multiplicative |  | 155.1 | 144.1 | Multiplicative |  | 8.5 | 8.8 | Additive |
|  | District 1 | 18.1 | 18.4 | Additive |  | 63.0 | 59.8 | Multiplicative |  | 4.6 | 4.6 | Additive |
|  | District 4 | 14.6 | 14.3 | Multiplicative |  | 71.2 | 65.4 | Multiplicative |  | 4.6 | 4.5 | Multiplicative |

**Figure S1: Description of confirmed malaria cases for each health district in children < 5 years old (A), in children ≥ 5 years old and adults (B), and in pregnant women (C) before and after imputation by spline interpolation, from 2008 to 2017 in Togo.** Health regions and target groups have different Y-axis scales.

**
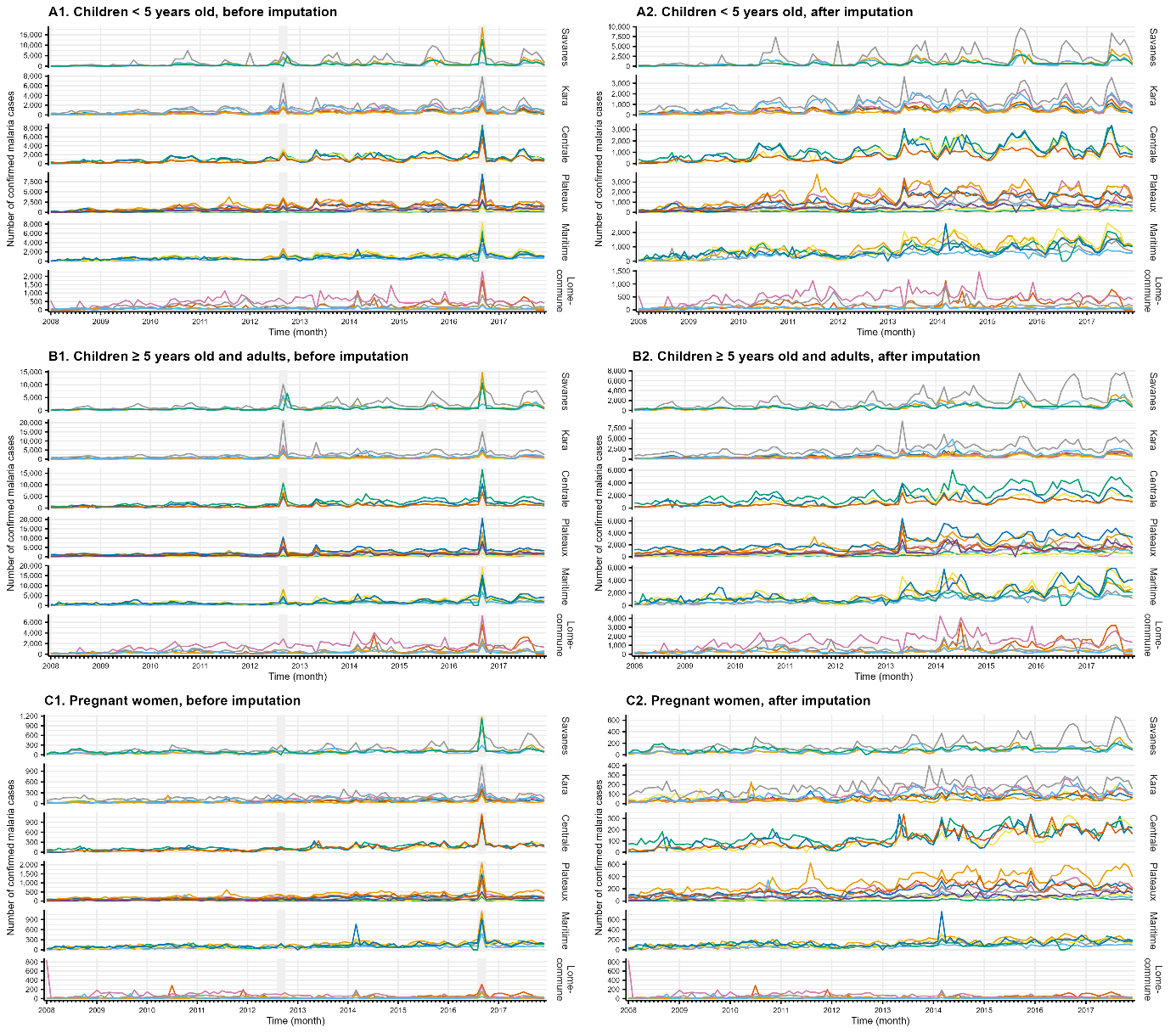
**

**Figure S2: Autocorrelograms of residuals estimated by the decomposition procedure for each health district in children < 5 years old (A), in children ≥ 5 years old and adults (B), and in pregnant women (C).** Abbreviation: ACF, autocorrelation function.


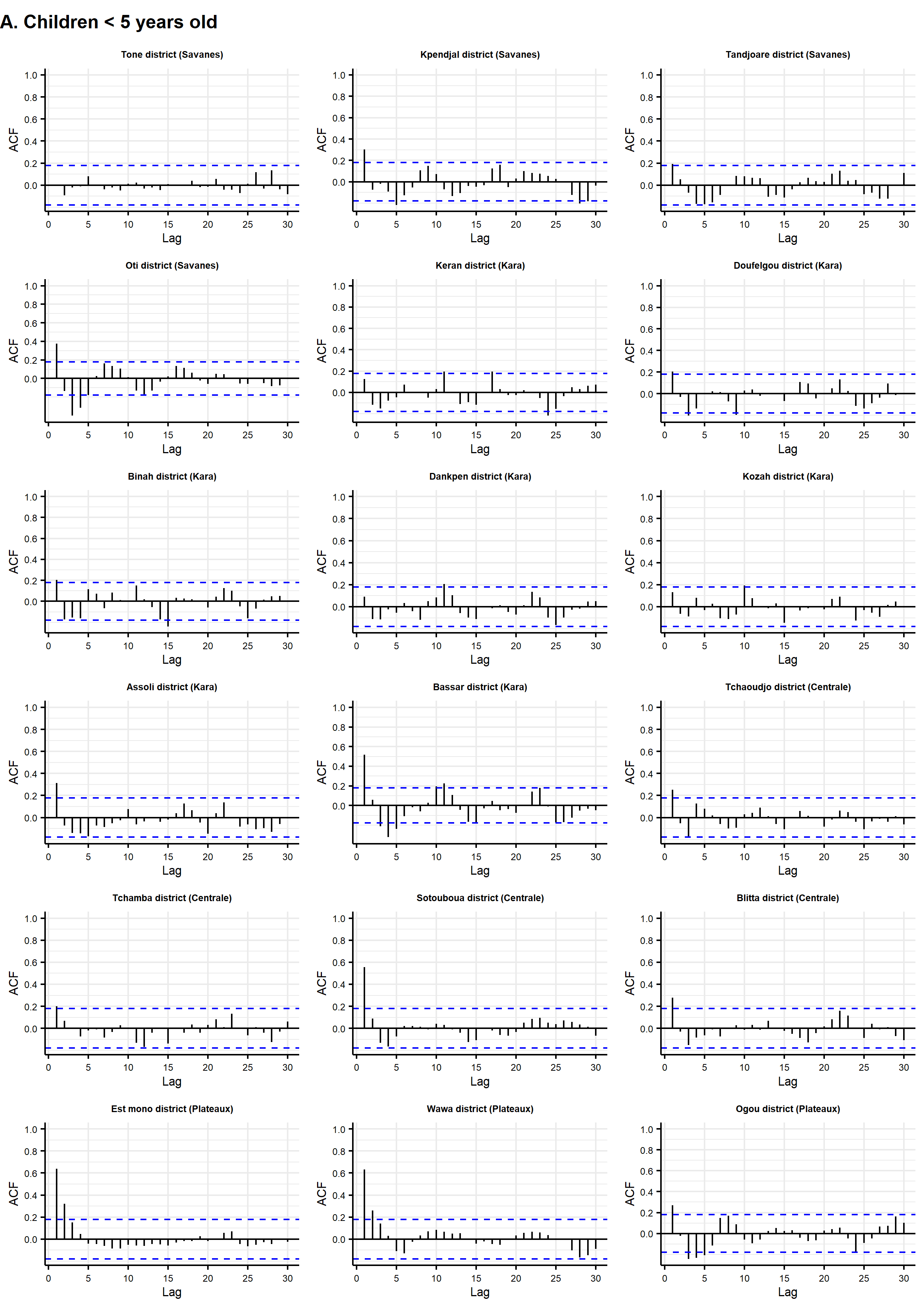


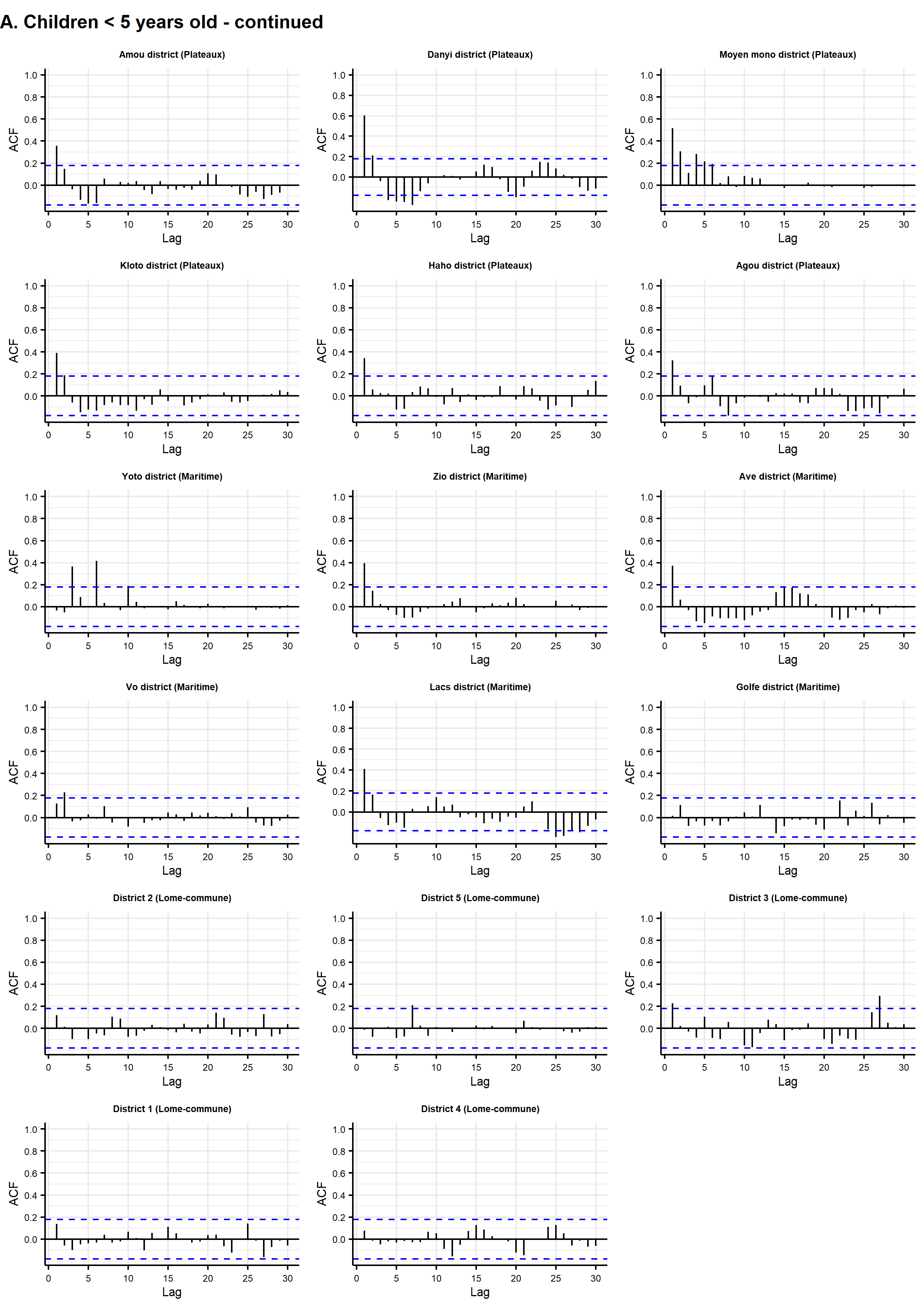


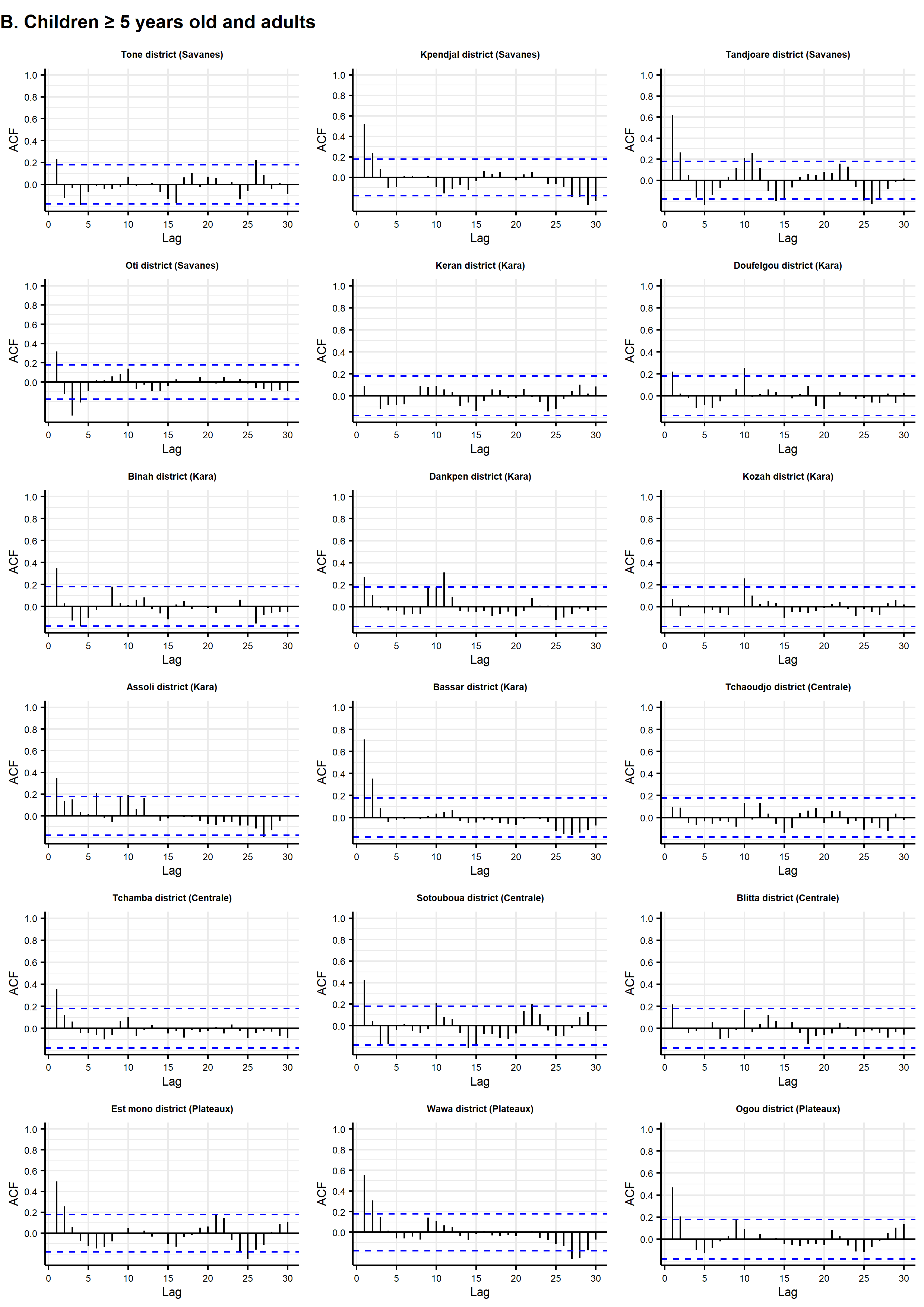


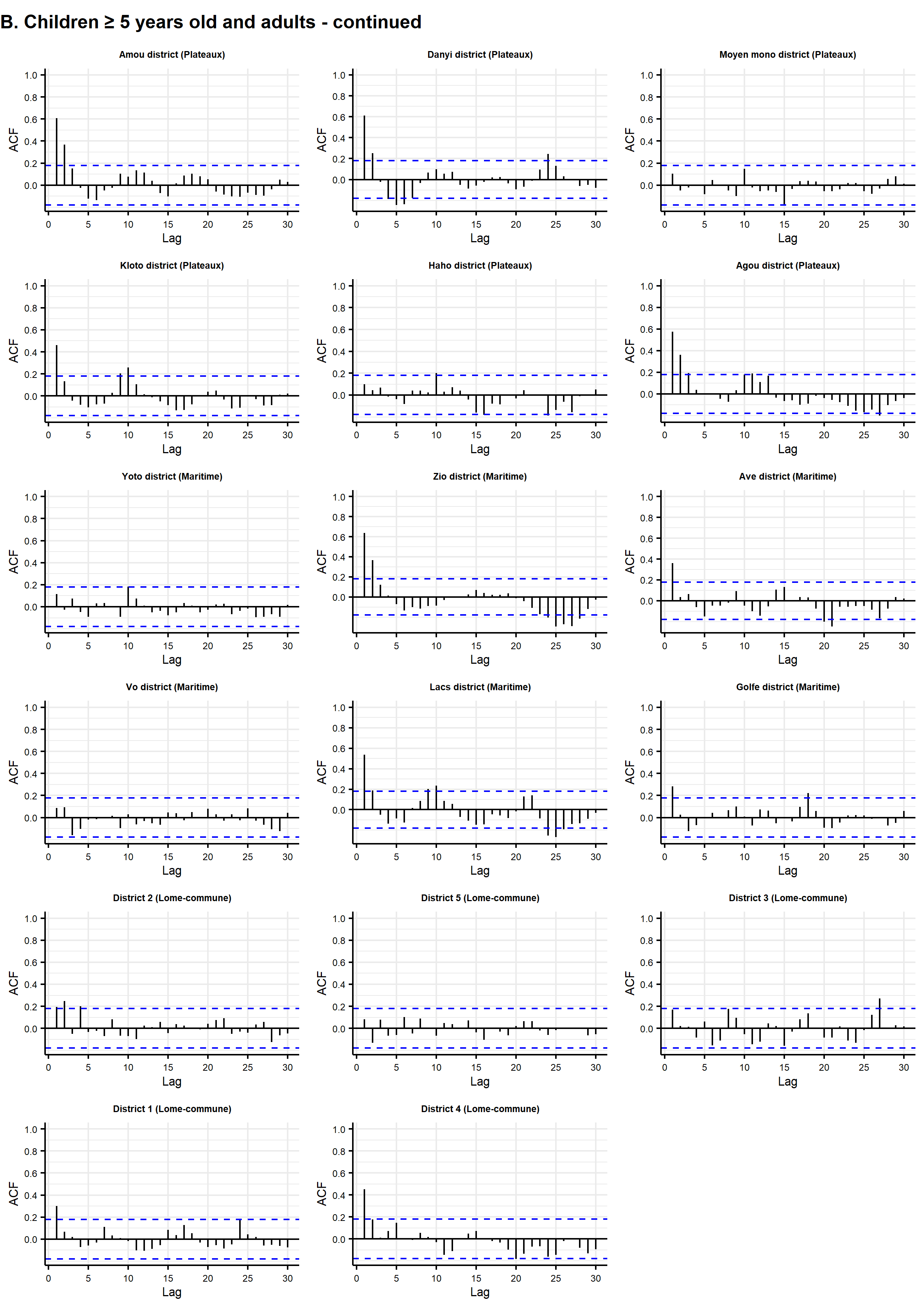


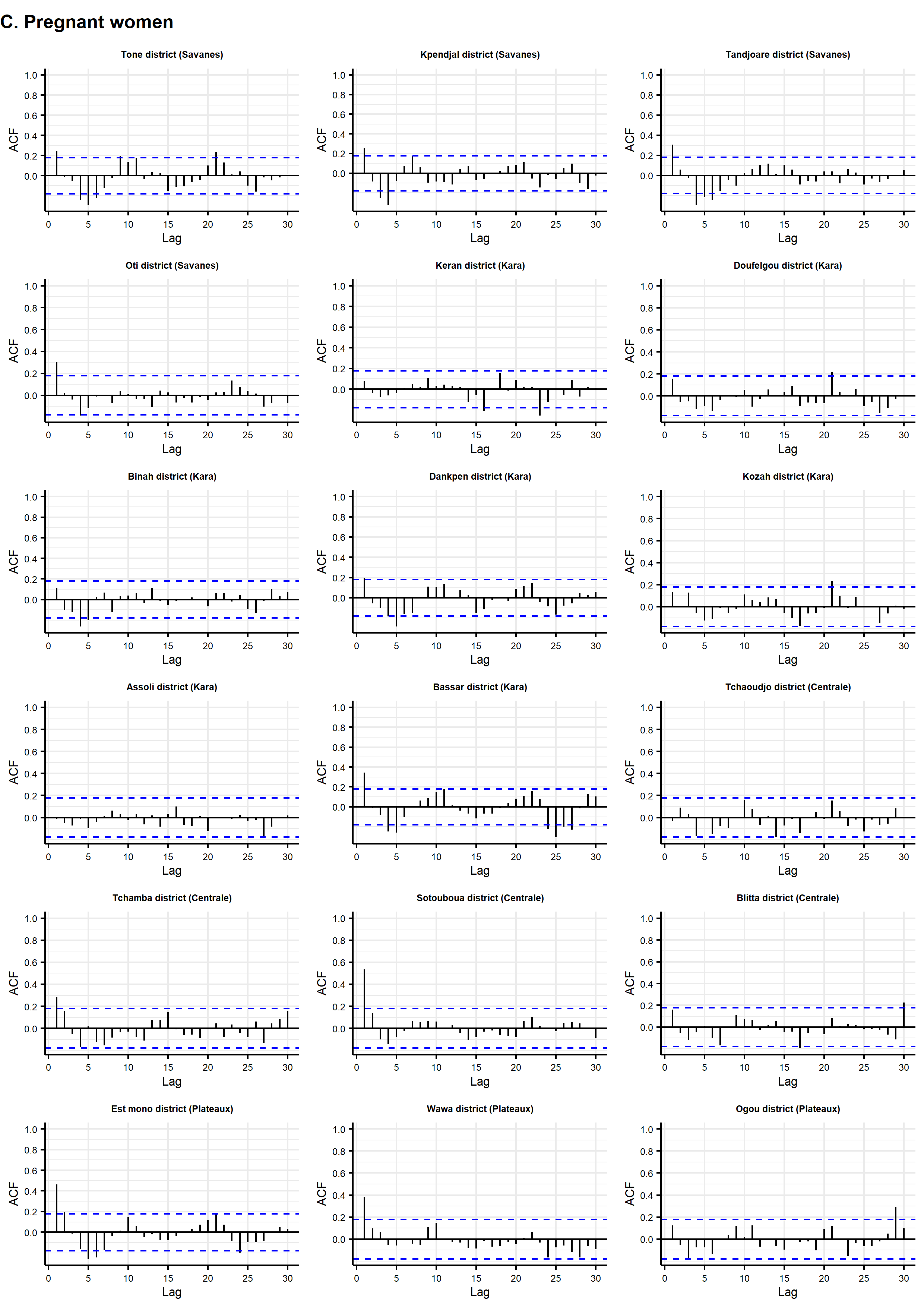


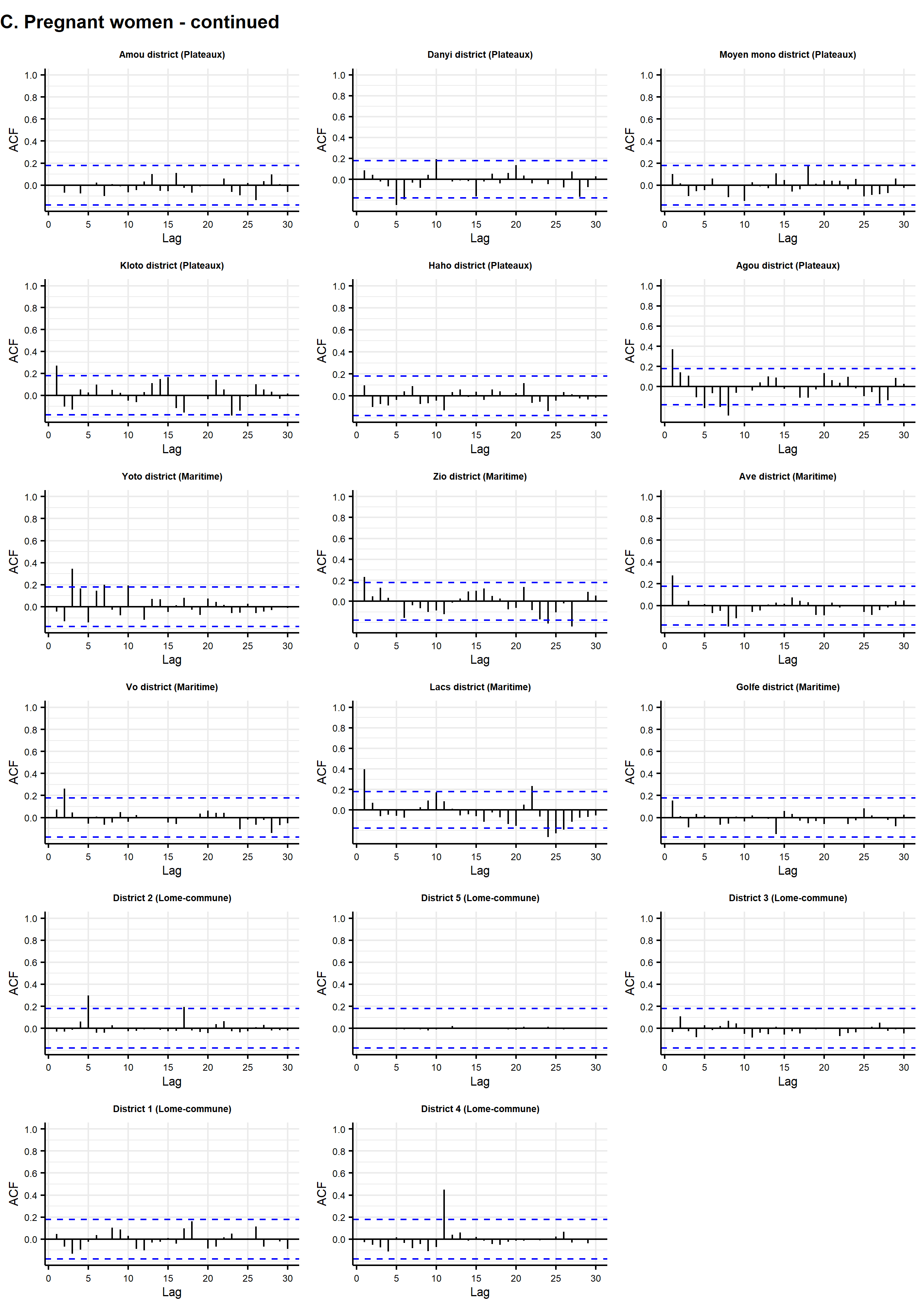


**Figure S3: Change of the number of confirmed malaria cases according to the month of the year during the period 2008-2017 in Togo.** The box plots represent the distribution of the number of cases for the decade studied, stratified by month. Data are grouped by target group. Time series have different Y-axis scales.


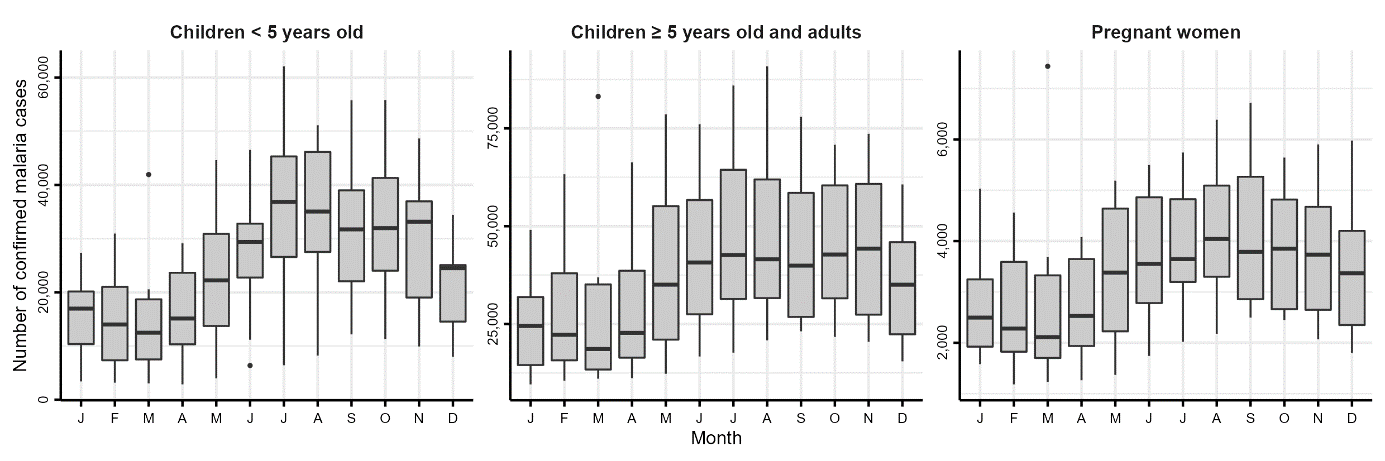


**Figure S4: Change of the number of confirmed malaria cases according to the month of the year during the period 2008-2017 in Togo.** The box plots represent the distribution of the number of cases for the decade studied, stratified by month. Each health district in children < 5 years old (A), in children ≥ 5 years old and adults (B), and in pregnant women (C) are presented. Time series have different Y-axis scales.


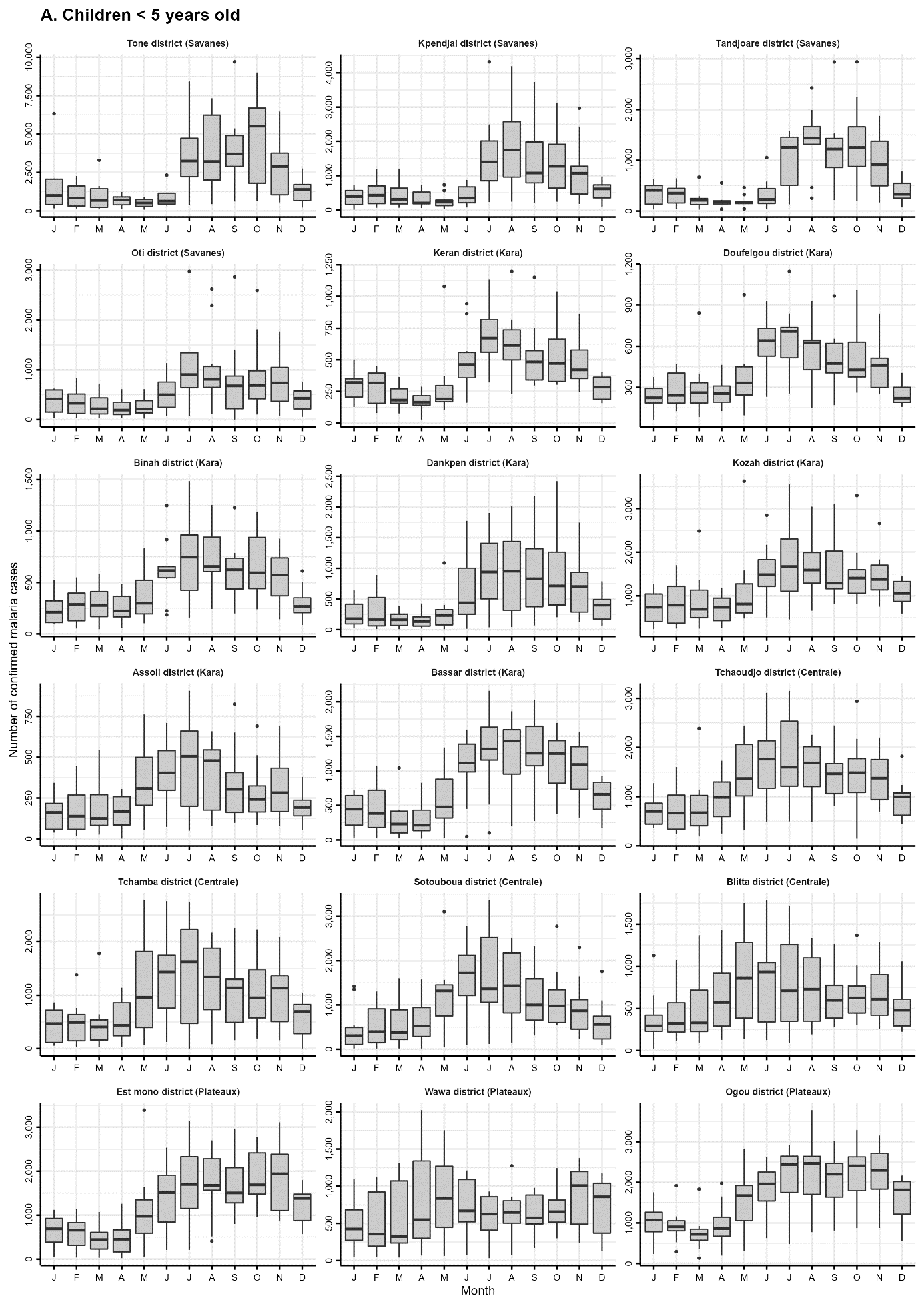


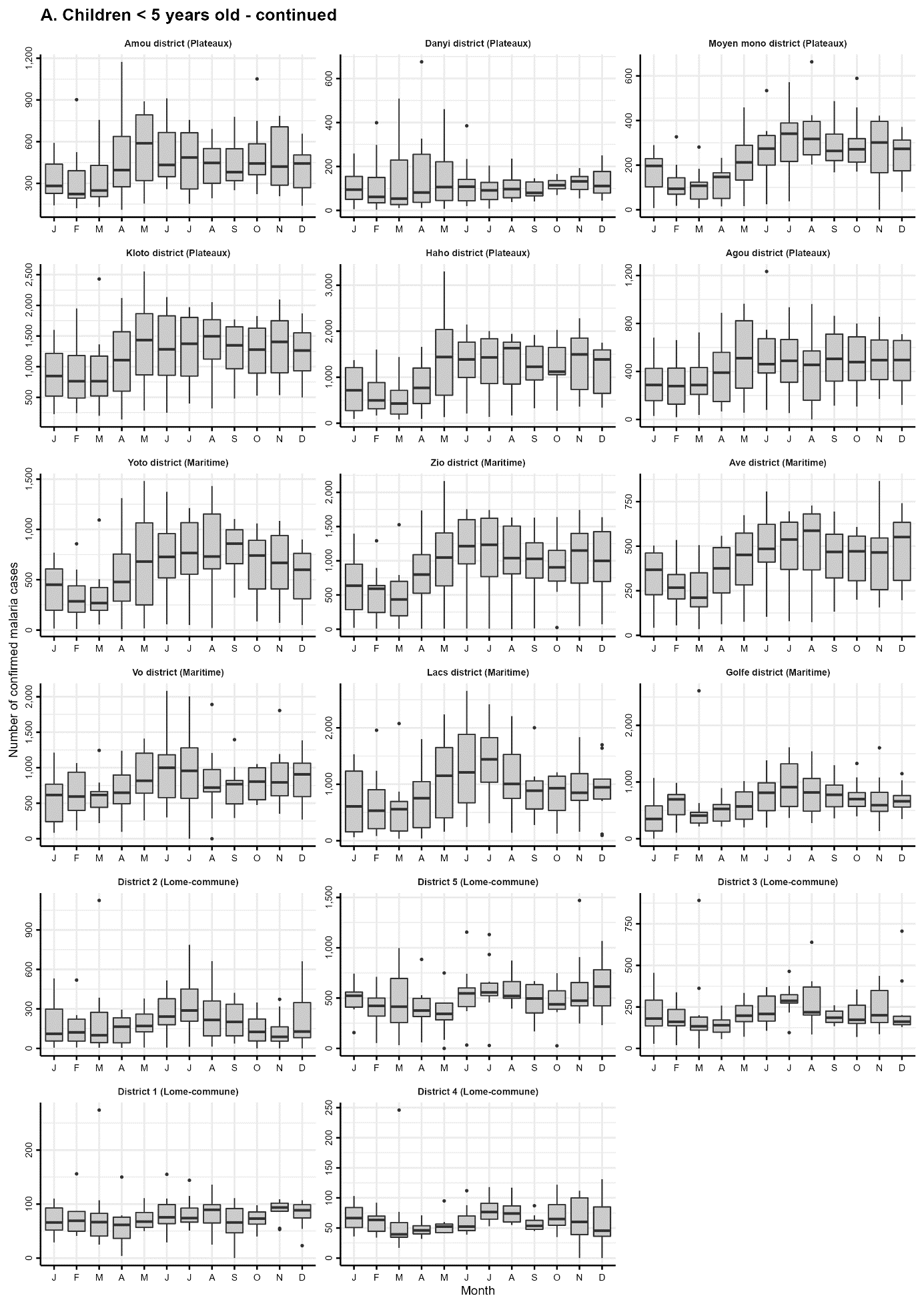


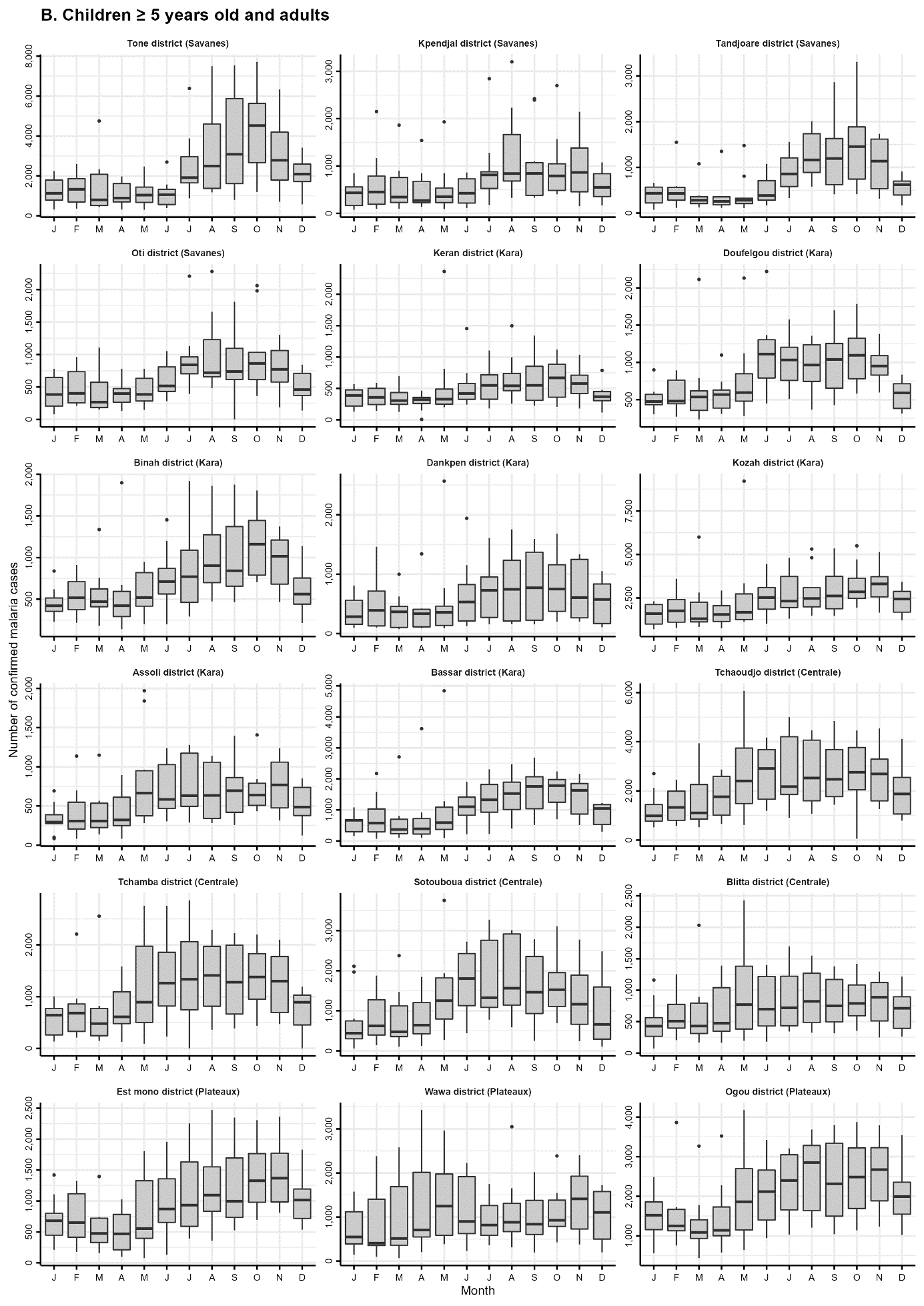


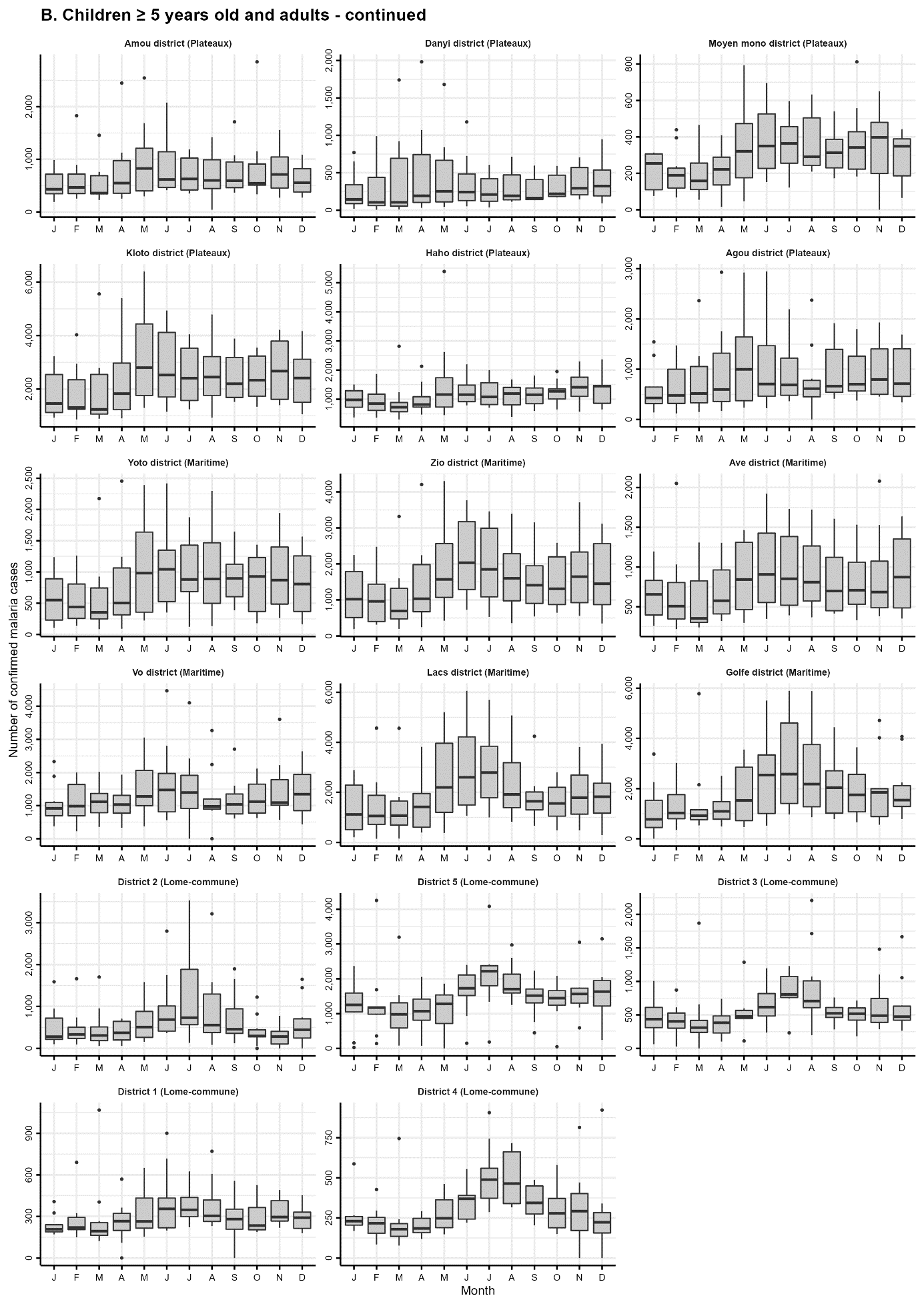


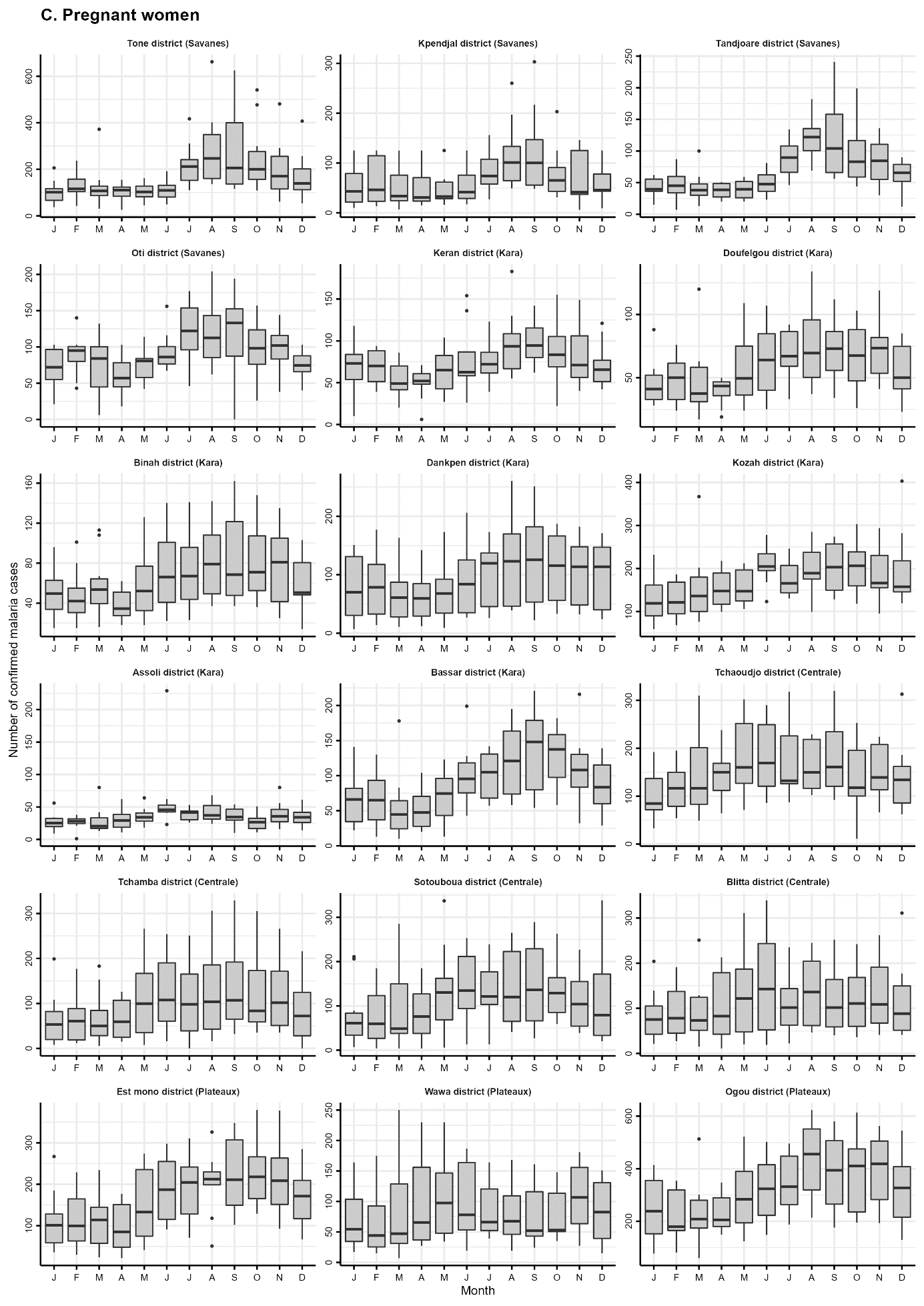


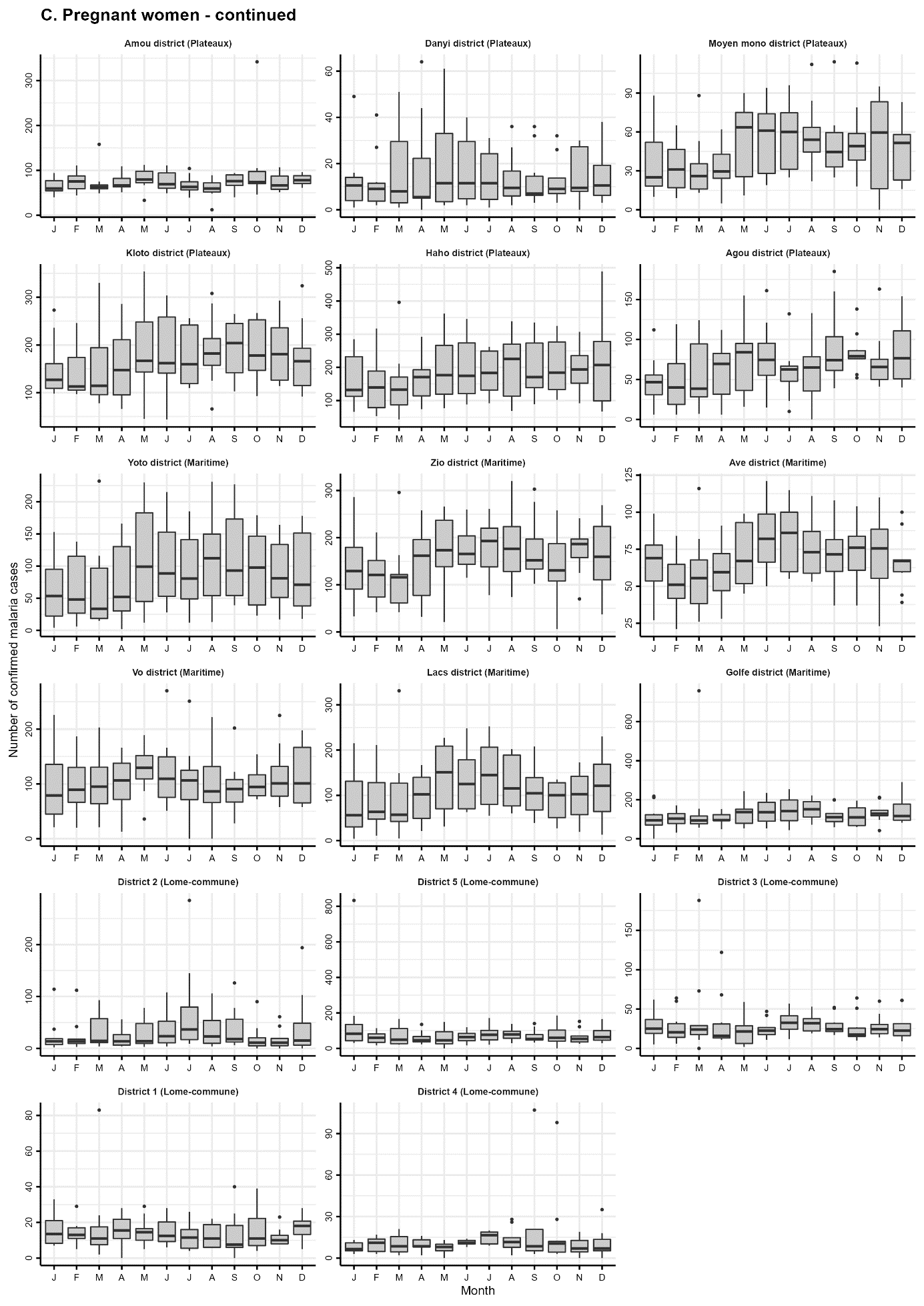


**Figure S5: Results of different imputation strategies of the values of the two artificial peaks on seasonal component estimated by the decomposition procedure.** Each health district in children < 5 years old (A), in children ≥ 5 years old and adults (B), and in pregnant women (C) are presented. Imputation by spline interpolation (main analysis), imputation by last observation carried forward corresponds, and no imputation (original data) corresponds to the grey, orange and red curves, respectively. Filled dots indicate maximum seasonal indices and empty dots indicate minimum seasonal indices. Time series have different Y-axis scales.


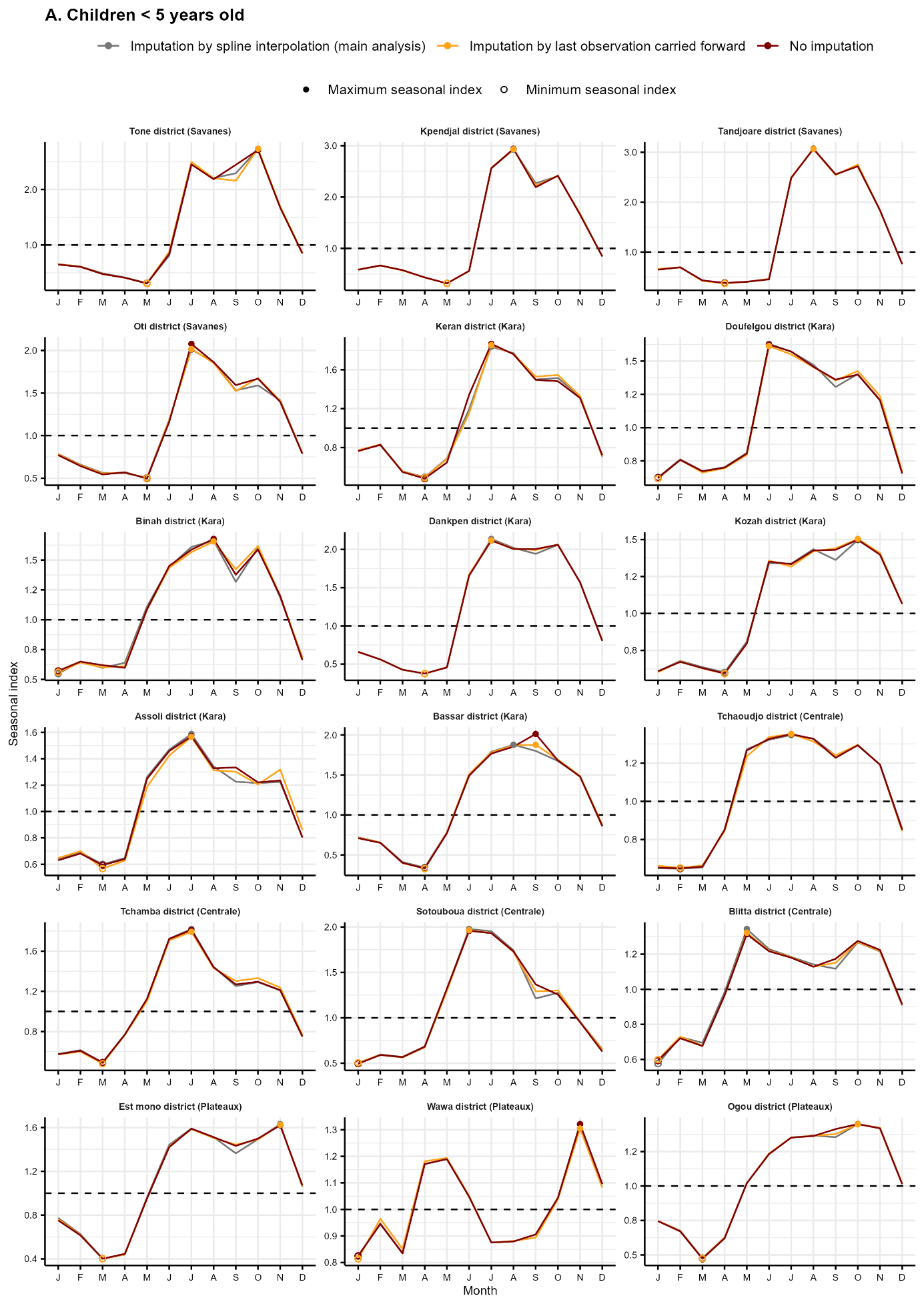


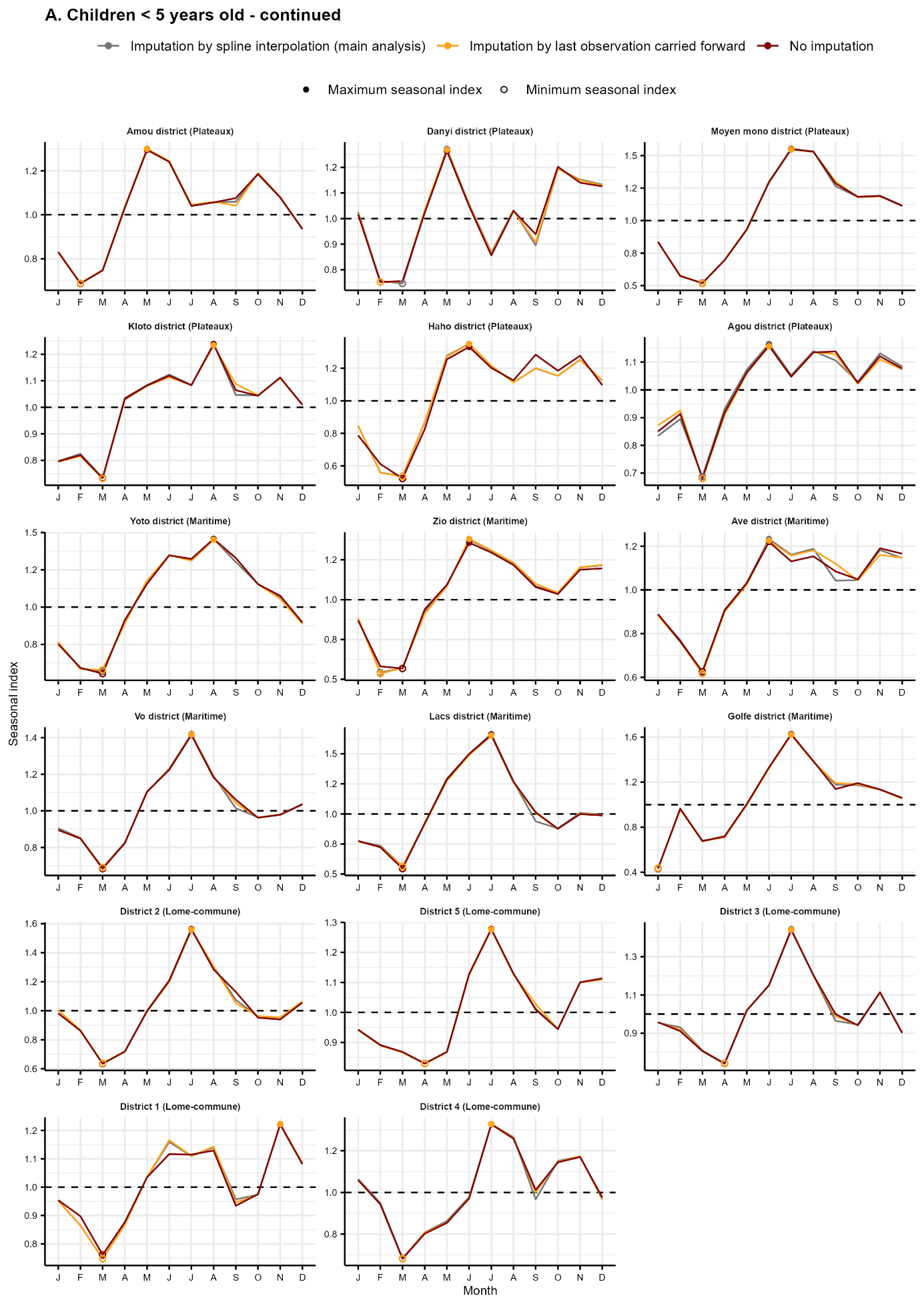


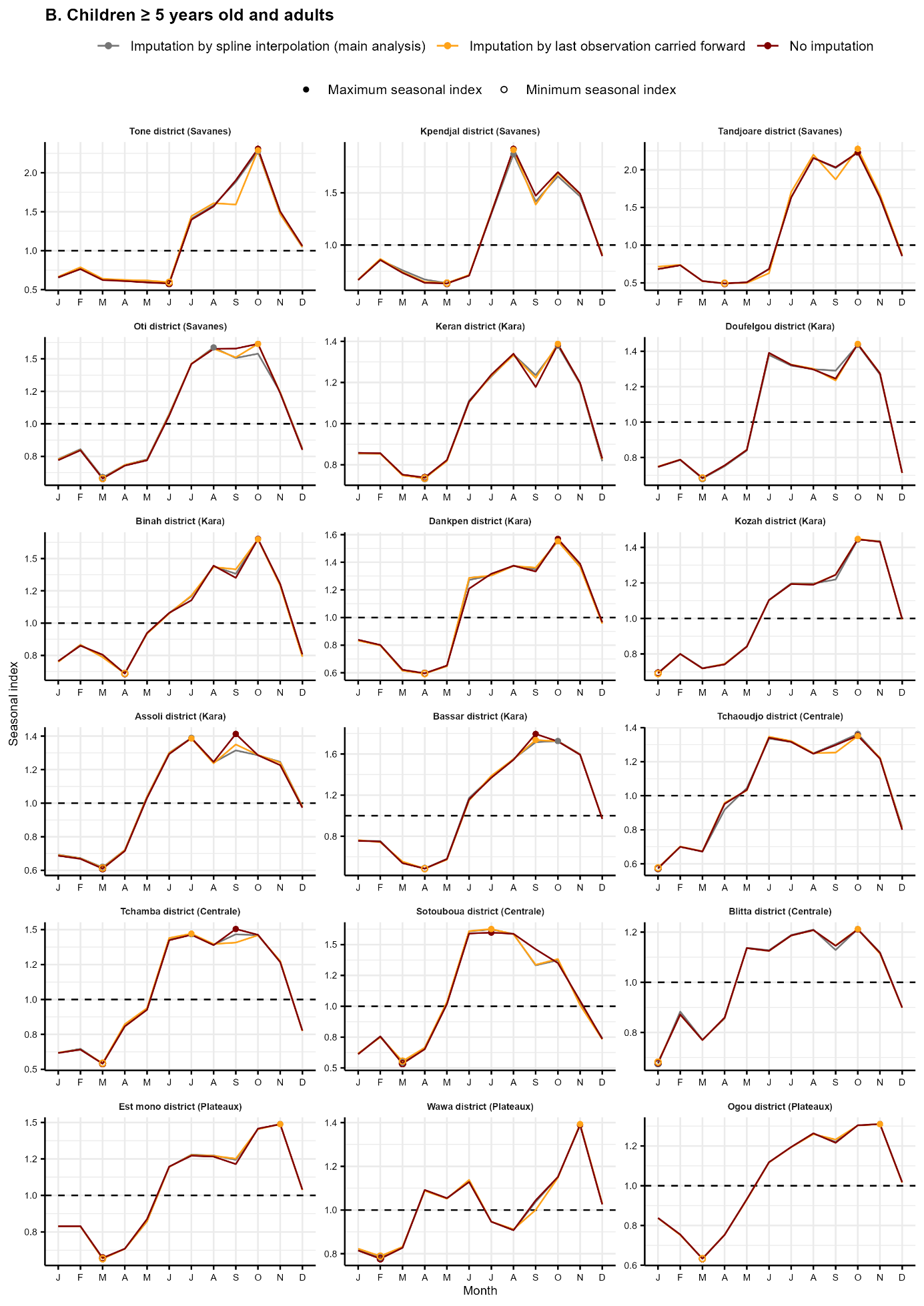


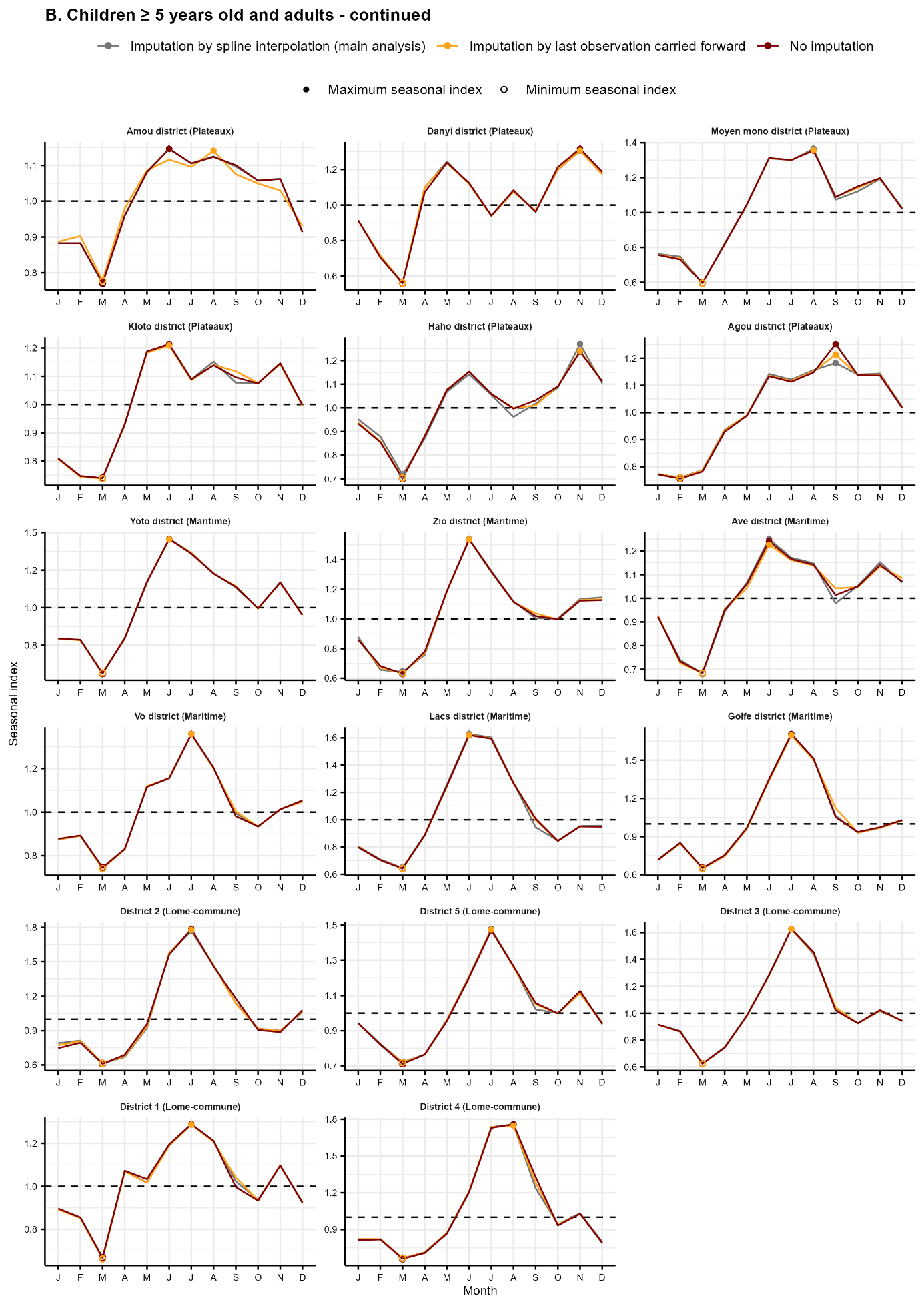


**
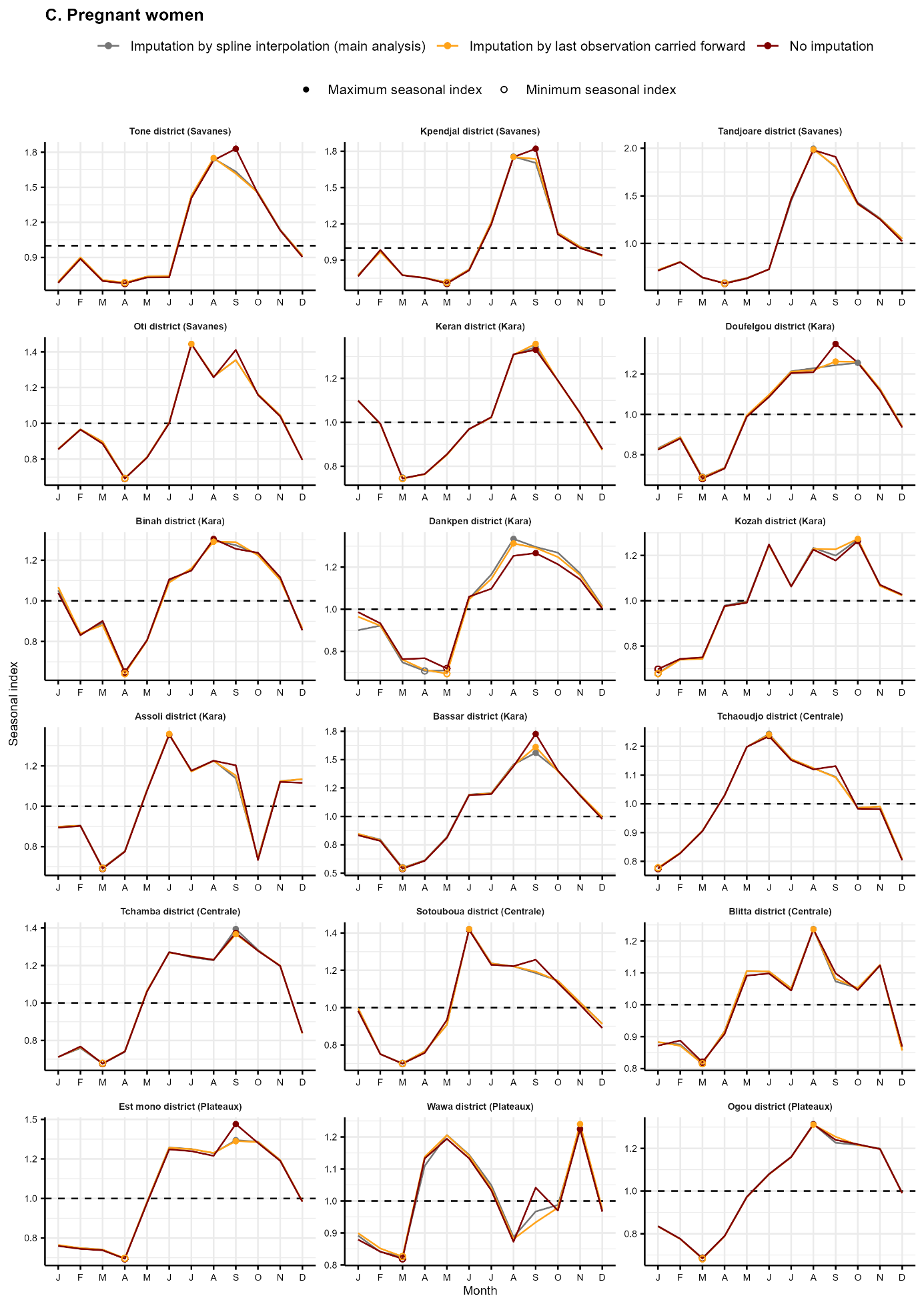
**

**
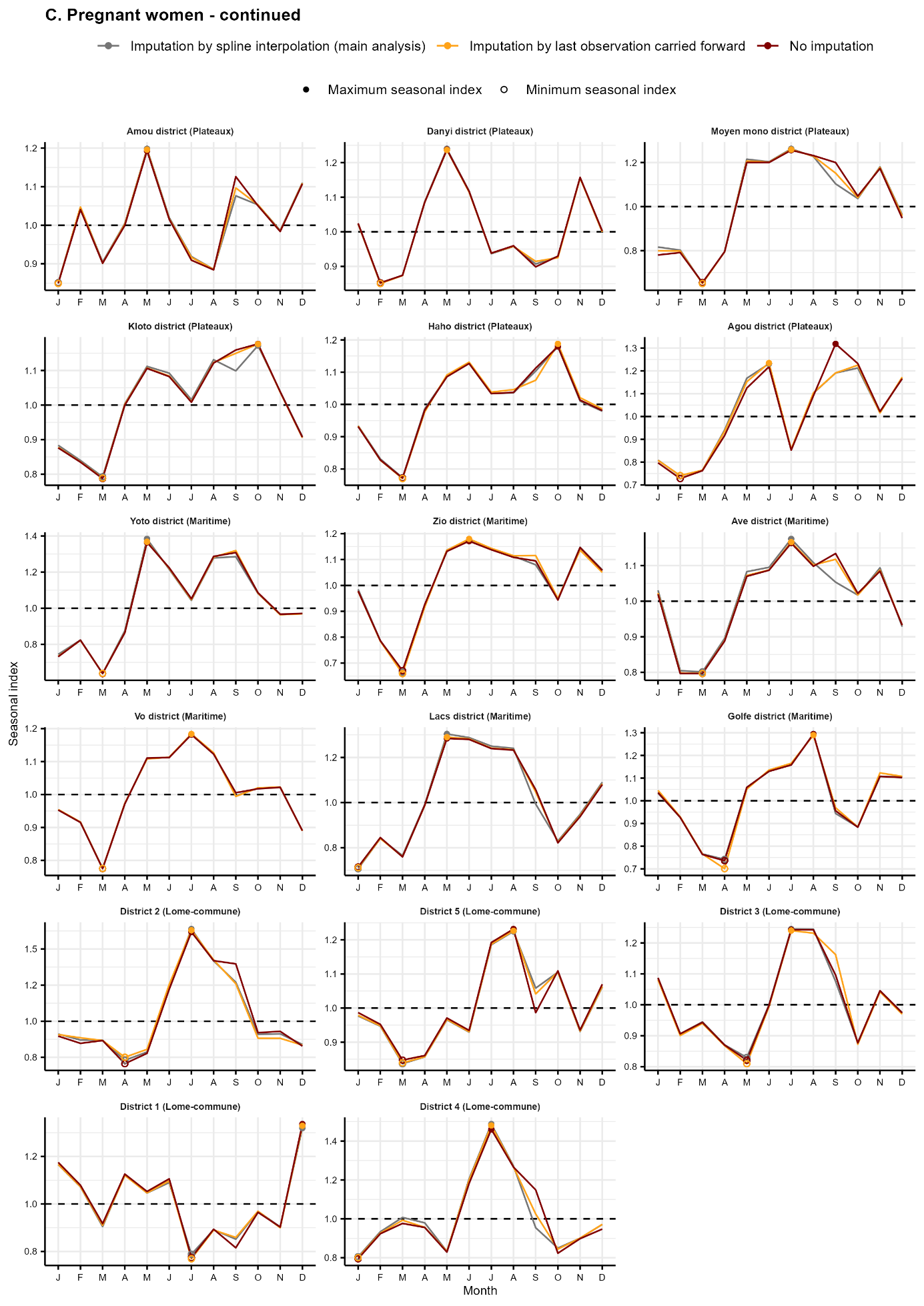
**
